# Impact of *Pseudomonas aeruginosa* biofilm exopolysaccharide composition on bacteriophage and bacteriophage-antibiotic combination activity

**DOI:** 10.1128/aac.00925-25

**Published:** 2025-12-05

**Authors:** Sean R. Van Helden, Callan R. Bleick, Dana J. Holger, Amer El Ghali, Jose Alexander, Keith S. Kaye, Steven H. Marshall, Laura J. Rojas, Robert A. Bonomo, Susan M. Lehman, Michael J. Rybak

**Affiliations:** 1 Anti-Infective Research Laboratory, Department of Pharmacy Practice, Eugene Applebaum College of Pharmacy and Health Sciences, Wayne State University 2954, Detroit, Michigan, USA; 2 Department of Pharmacy Practice, Barry and Judy Silverman College of Pharmacy, Nova Southeastern University 2814, Fort Lauderdale, Florida, USA; 3 University of New Mexico College of Pharmacy 15520, Albuquerque, New Mexico, USA; 4 Department of Microbiology, Virology and Immunology, AdventHealth Central Florida 558924 , Orlando, Florida, USA; 5 Department of Medicine, Rutgers Robert Wood Johnsons Medical School, New Brunswick, New Jersey, USA; 6 Louis Stokes Cleveland Department of Veterans Affairs Medical Center 20083, Cleveland, Ohio, USA; 7 Department of Molecular Biology and Microbiology, Case Western Reserve University School of Medicine 12304, Cleveland, Ohio, USA; 8 CWRU-Cleveland VAMC Center for Antimicrobial Resistance and Epidemiology (Case VA CARES) Cleveland, Cleveland, Ohio, USA; 9 Departments of Medicine, Pharmacology, Biochemistry, and Proteomics and Bioinformatics, Case Western Reserve University School of Medicine 12304, Cleveland, Ohio, USA; 10 Center for Biologics Evaluation and Research, US Food and Drug Administration 67149, Silver Spring, Maryland, USA; 11 Department of Pharmacy Services, Detroit Receiving Hospital, Detroit Medical Center 2956, Detroit, Michigan, USA; 12 Division of Infectious Diseases, School of Medicine, Wayne State University 2954, Detroit, Michigan, USA; Shionogi Inc., Florham Park, New Jersey, USA

**Keywords:** PDR, XDR, DTR, MDR, multidrug resistance, medical device infections, biofilm matrix, biofilm, *Pseudomonas aeruginosa*, antimicrobial resistance

## Abstract

Multidrug-resistant *Pseudomonas aeruginosa* is a leading cause of hospital-acquired infections, including medical device infections, partially due to the organism’s ability to produce biofilm on prosthetic material. Increased antibiotic tolerance of bacteria within biofilm, along with the increasing prevalence of infections caused by multidrug-resistant *P. aeruginosa*, including extensively drug-resistant phenotypes, results in scenarios where conventional antibiotics may fail to effectively treat these infections. Bacteriophage (phage) therapy is a promising alternative to conventional antimicrobial therapy that can provide potent antibiofilm activity, particularly when combined with antibiotics. The impact of *P. aeruginosa* biofilm phenotype on the efficacy of phage-antibiotic combinations remains unclear. We characterized the biofilms of a panel of 10 clinical and two laboratory *P. aeruginosa* isolates by determining the minimum biofilm inhibitory concentrations, biofilm production capabilities, and phage activity. Furthermore, we quantified the exopolysaccharide content of *P. aeruginosa* strains PAO1 and PA14 and confirmed they produced Class II and Class I biofilm, respectively. We then selected a triple-phage cocktail with broad activity across all *P. aeruginosa* strains for evaluation of antibiofilm activity against PAO1 and PA14 *in vitro*. Through spectrophotometric growth suppression and time-kill analyses, we found the triple-phage cocktail + ciprofloxacin to be more efficacious in biofilm eradication than either modality alone. Phage 109 displayed potent antibiofilm activity against both strains irrespective of planktonic activity, whereas phages E2005-C and EM-T3762627-2_AH displayed distinct antibiofilm activity based on biofilm phenotype. These data warrant further investigation into the impact of *P. aeruginosa* biofilm phenotype on phage antibiofilm activity.

## INTRODUCTION

Healthcare-associated infections pose a significant health risk for inpatients. The Centers for Disease Control and Prevention estimates that there are over 1.7 million healthcare-associated infections annually, 35% of which are deemed medical device infections (MDIs) (1). *Pseudomonas aeruginosa* is a leading cause of hospital-acquired bloodstream infections, pneumonia, and device-associated infections, all of which are associated with high morbidity and mortality (1). This is compounded by the increasing incidence of infections due to multidrug-resistant (MDR) *P. aeruginosa* strains, including difficult-to-treat resistance (DTR) and extensively drug-resistant (XDR) phenotypes (2). Additionally, these isolates can be particularly difficult to treat due to their ability to form extensive biofilm on foreign material. Limited antimicrobial penetration into the biofilm matrix, differences in metabolic activity of bacterial cells between different biofilm layers, drug tolerance, and increased resistance gene expression in biofilm-embedded cells are among the fundamental causes of subsequent treatment failure (3–5). Consequently, *P. aeruginosa* biofilms display increased minimum inhibitory concentrations (MICs) when in biofilm compared to their planktonic counterparts (6). These factors collectively result in situations where nearly all conventional antibiotics may be ineffective against MDR *P. aeruginosa* MDIs.

*P. aeruginosa* biofilms are comprised of four primary components: polysaccharides, proteins, extracellular DNA (eDNA), and lipids (7). The exopolysaccharides Psl, Pel, and alginate encompass much of the biofilm architecture. Non-mucoid *P. aeruginosa* biofilms are primarily comprised of Psl and Pel, whereas mucoid strains predominantly produce alginate in excess. The structure of Psl is comprised of D-mannose, D-glucose, and L-rhamnose, while Pel is composed of N-acetylgalactosamine and N-acetylglucosamine (8, 9). The quantity of Psl and Pel present within the biofilms of non-mucoid *P. aeruginosa* varies considerably (10). Colvin et al. proposed four phenotypic categories of *P. aeruginosa* biofilm based on its exopolysaccharide composition: Class I) Pel-rich, Class II) Psl-rich biofilms, Class III) balanced expression of both Pel and Psl, and Class IV) matrix over-producers (10). These exopolysaccharides, along with eDNA, make up the biofilm architecture responsible for bacterial resistance to antibiotics (7). The biofilm of laboratory standard strain PAO1 is rich in Psl and is phenotypically categorized as a Class II biofilm (10). Conversely, laboratory standard strain PA14, which carries a three-gene deletion in the *psl* operon that prevents Psl production, solely produces Pel in its biofilm and is a representative example of a Class I biofilm (11, 12).

Bacteriophages (phages) are viruses that infect and utilize the cellular machinery of bacteria to replicate (13). Lytic phages are of particular interest due to their ability to lyse bacterial cells to release progeny that will infect and kill neighboring cells. Leveraging this property has proven efficacious in both *in vitro* and *in vivo* experiments, with potentially promising results in some clinical case reports and structured clinical trials (6, 14–17). However, considerable uncertainty persists regarding the optimal selection and application of phages to effectively target certain bacterial communities. For example, phages have been studied as both adjuncts and alternatives to antibiotic therapy (18–20). *In vitro* data suggest that phage-antibiotic combination (PAC) therapy can be more efficacious than either modality alone in eradicating planktonic bacteria, as well as bacterial biofilms (6, 17, 21–23), but the basis for predicting optimal combinations is largely unknown. Some phage species produce enzymes known as phage depolymerases, which degrade the exopolysaccharide present within biofilms (24). Phage depolymerases demonstrate substrate specificity, suggesting they may preferentially target particular biofilm types while exhibiting minimal effect on others. While the use of PAC therapy has been shown to successfully eradicate *P. aeruginosa* biofilms *in vitro*, the influence of biofilm composition on phage activity remains poorly understood (6). In this study, we evaluate the impact of *P. aeruginosa* biofilm phenotype on the efficacy of a previously validated three-phage cocktail alone and in combination with ciprofloxacin (CIP).

## RESULTS

### Phage activity and bacterial antibiotic susceptibility

**Fig 1:**
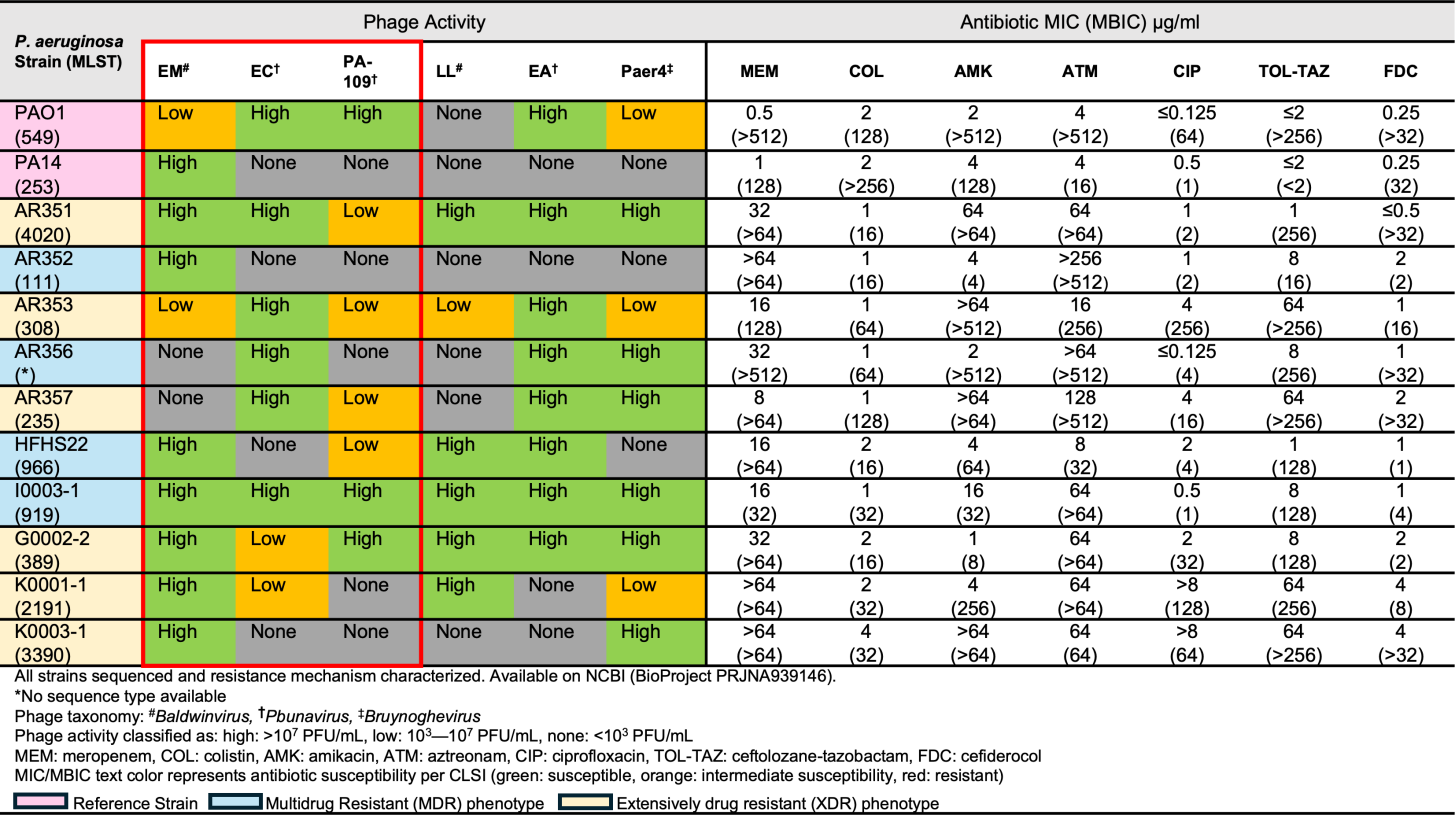
Phage activity and antibiotic susceptibilities of 10 clinical MDR/XDR *P. aeruginosa* strains and two laboratory standard reference strains.

Purified 1 × 10^7^ PFU/mL phage stocks of six phages—EM, EC, 109, LL, EA, and Paer4—were evaluated for activity via agar plaque assay against a panel of 10 well-characterized clinical MDR/XDR *P. aeruginosa* strains, as well as two laboratory standard reference strains (PAO1 and PA14) in their planktonic form (25). As seen in Fig. 1, phages EM, EC, and EA had high activity against a majority of clinical strains tested. Interestingly, EM was the sole phage that exhibited activity against PA14 and AR352. While 109 appeared to demonstrate limited activity against many clinical strains, previous data regarding its impact on *P. aeruginosa* biofilm suggest that this phage exhibits significant antibiofilm activity compared to other phages in our collection when added to phage cocktails (6, 26). Phage Paer4 did not have substantial activity against PAO1, despite recent reports that a related phage uses Psl as a receptor (27). Additionally, the MICs of all *P. aeruginosa* strains were evaluated against a variety of anti-pseudomonal antibiotics (Fig. 1). The minimum biofilm inhibitory concentrations (MBICs) of these antibiotics were assessed using the previously reported pin-lid technique (28). In nearly all instances, MBIC values greatly exceeded their corresponding MIC values, signifying heightened antibiotic tolerance of bacteria within biofilms.

### Biofilm quantification

**Fig 2:**
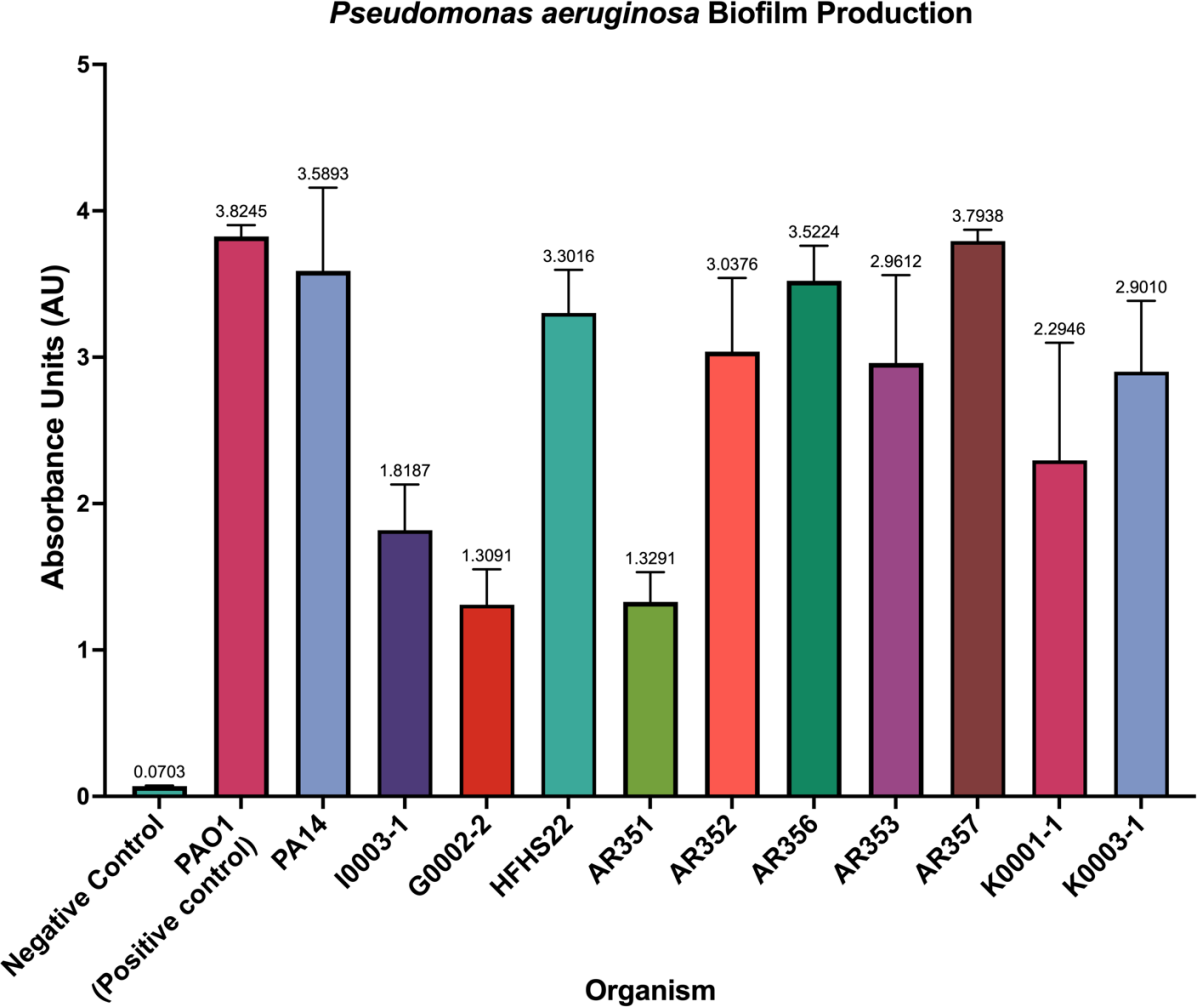
Biofilm quantification of *P. aeruginosa* strains of interest reported using absorbance units at OD_570_. Negative control indicates lack of organisms present. PAO1 functioned as a positive control. Error bars represent standard deviation. All experiments were performed in triplicate.

Biofilm production varies considerably across *P. aeruginosa* strains due to genotypic variation in biofilm regulator and quorum-sensing genes, among other factors (7, 29). Using spectrophotometric quantification assays, we determined that laboratory strains PAO1 and PA14 produced comparable levels of biofilm (3.82 ± 0.08 vs 3.59 ± 0.57 absorbance units at 570 nm [AU_570_]), consistent with their established biofilm-forming capabilities (Fig. 2). Among the clinical isolates, AR356 and AR357 demonstrated the highest biofilm production (3.52 ± 0.24 and 3.79 ± 0.08 AU_570_), while I0003-1, G0002-2, and AR351 produced the least (1.82 ± 0.31, 1.31 ± 0.24, and 1.33 ± 0.20 AU_570_), indicating potential phenotypic differences that may impact antibiotic tolerance and persistence in clinical settings. These findings underscore the heterogeneity of biofilm-forming ability among *P. aeruginosa* strains, which may have implications for phage and phage-antibiotic combination activity.

### Exopolysaccharide quantification

**Fig 3:**
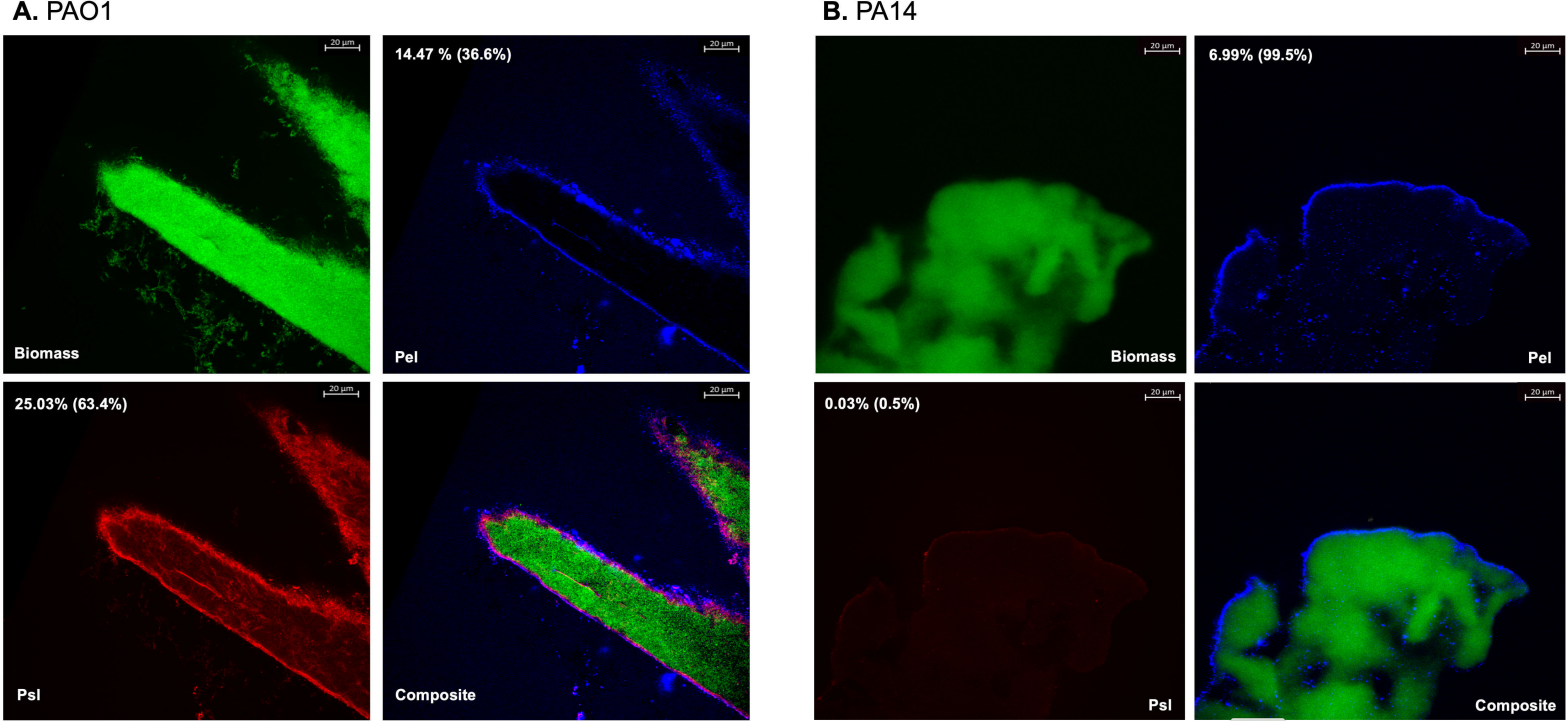
Pel and Psl content of 96 h biofilms of (**A**) *P. aeruginosa* strain PAO1 and (**B**) *P. aeruginosa* strain PA14 stained with Psl-specific lectin (HHA, blue), Pel-specific lectin (WFL, red), and general biomass stain (Syto62, green). Images comprise single optical planes at 40× magnification. Percentages represent the proportion of the total biomass area attributed to each specific exopolysaccharide, with the percentage of each exopolysaccharide relative to the total exopolysaccharide area indicated in parentheses.

To investigate the effect of *P. aeruginosa* biofilm exopolysaccharide composition on phage activity, we sought to quantify the amount of Psl and Pel present within the biofilm of PAO1 and PA14 (Fig. 3). These two strains were selected due to the prior characterization of their biofilm phenotypes in the literature, allowing for validation of the phenotyping method employed in this study (10). Utilizing a fluorescent staining technique developed by Jennings et al. and subsequent pixel analysis, it was determined that the biofilm of PAO1 comprised 25% Psl and 14% Pel, relative to total biomass, corroborating its previous designation as a Class II biofilm (9, 10). Conversely, the biofilm of PA14 contained 0% Psl and 7% Pel, relative to biomass, consistent with the established mutation in its *psl* operon. These findings indicate that PA14 expresses a Class I biofilm phenotype. These findings align with the previously published phenotypes of these two strains, suggesting that the exopolysaccharide quantification technique employed in this study may be utilized for phenotyping of additional strains in future studies.

### Spectrophotometric analysis of phage-antibiotic activity against established biofilms

**Fig 4:**
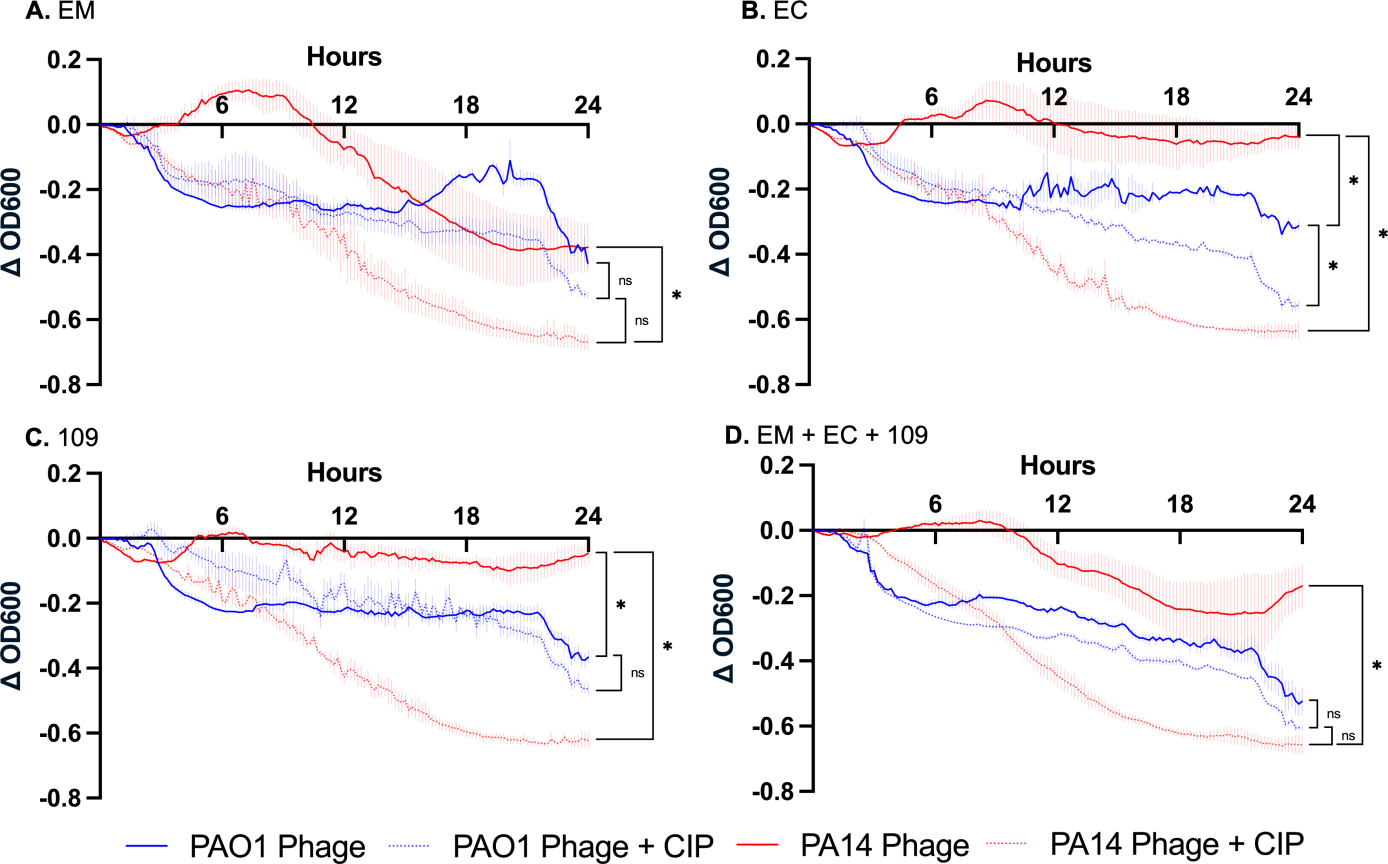
Twenty-four-hour continuous relative biofilm growth suppression following a 24 h biofilm maturation phase. Data are presented as the difference in optical density at 600 nm (OD_600_) values relative to the growth control after normalization to starting OD_600_ to account for differences in biofilm production between strains. Each plot illustrates the growth of PAO1 and PA14 treated with respective phage ± CIP relative to growth control. Phages were dosed at MOI ≅ 1, and CIP was dosed at 0.5× MIC for each organism. Wells with uninoculated media were utilized as negative controls. Lines represent the mean of triplicate experiments, and error bars represent the standard error of the mean. Asterisks denote significant (*P* < 0.05) differences between treatments of interest, while “ns” signifies non-significance. Statistical analysis consisted of a one-way ANOVA, followed by Tukey’s HSD *post hoc* test for multiple comparisons. Denoted statistical tests displayed within the figure are non-exhaustive. Full statistical analysis is available in the supplementary material. (**A**) Phage EM. (**B**) Phage EC. (**C**) Phage 109. (**D**) Triple cocktail of phages EM, EC, and 109.

Phages EM and EC were selected for further evaluation due to their complementary planktonic activity across all strains evaluated in Fig. 1. Moreover, phages EM and EC are taxonomically categorized as *Baldwinvirus* and *Pbunavirus*, respectively, providing species diversity to potential phage cocktails and possibly contributing to their differing activity profiles. Additionally, phage 109 was selected for its previously noted antibiofilm activity (6). A triple-phage cocktail combining EM, EC, and 109 with CIP was previously studied in a 4 day CDC biofilm reactor model with strain AR351, resulting in a bacterial burden reduction of ≥5 log_10_ CFU/mL compared to the initial inoculum (6). Consequently, a cocktail of phages EM, EC, and 109 at an MOI = 1.0 was selected for further analysis in combination with CIP, which was chosen based on previous data suggesting improved efficacy against *P. aeruginosa* biofilms compared to other antibiotics (6, 30). Given the distinct biofilm phenotypes of PAO1 and PA14, phages were tested against 24 h biofilms of both strains, either alone or in combination with CIP at 0.5× MIC to simulate biofilm treatment failure scenarios (Fig. 4).

A one-way ANOVA confirmed a significant treatment effect on both PAO1 and PA14 (*F*(29, 58) = 24.2, *P* < 0.001, ω = 0.88). PA14 demonstrated greater resistance to phage treatment than PAO1 with phages EC and 109, while EM activity was similar across the two strains. Phage EM exhibited the highest activity against PA14 biofilms, whereas EC and 109 were most active against PAO1. The addition of CIP to phage drastically improved bacterial eradication in PA14, even in combination with EC and 109, which display minimal activity when administered as monotherapy. PA14 appears to be highly susceptible to phage + CIP combinations. The beneficial effect of adding CIP to phage appeared less pronounced against PAO1 biofilms. Interestingly, the triple-phage cocktail (without CIP) appeared to perform worse than EM monotherapy against PA14 (Fig. S1B), although this finding was not statistically significant (*P* = 0.280).

### Biofilm time-kill analyses (bTKAs)

**Fig 5:**
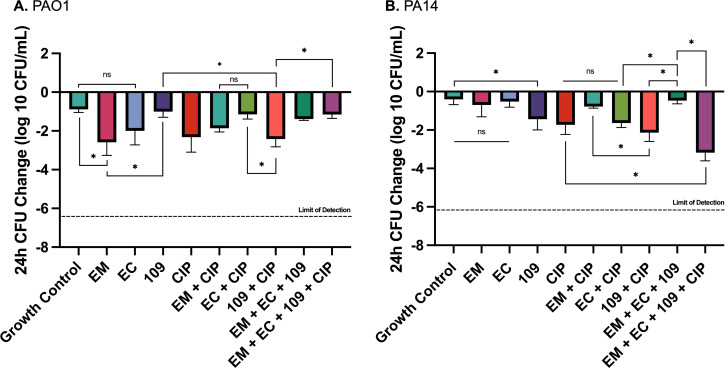
Twenty-four-hour biofilm time-kill analyses of *P. aeruginosa* laboratory strains (**A**) PAO1 and (**B**) PA14. Biofilms were formed on glass beads for 24 h prior to administration of the treatment regimen. Phages were dosed at MOI ≅ 1 when possible. For phages with low activity against the target bacterial strain, where titers against target organisms could not reach MOI ≅ 1, the highest possible MOI was used, provided it did not exceed 1. CIP was dosed at 0.5× MIC to simulate treatment failure conditions. Wells with uninoculated media were utilized as negative controls. Data represent the mean ± standard deviation of two biological replicates, each performed in technical duplicate. Asterisks denote significant (*P* < 0.05) differences between treatments of interest, while “ns” signifies non-significance. Statistical analysis consisted of a one-way ANOVA with Tukey’s HSD *post hoc* test for multiple comparisons. Denoted statistical tests displayed within the figure are non-exhaustive. Full statistical analysis is available in the supplementary material. Limits of detection were determined to be −6.39 and −6.13 Δlog_10_ CFU/mL for PAO1 and PA14, respectively.

To further investigate our spectrophotometric findings, previously validated bTKAs were performed utilizing identical phage and antibiotic combinations (6, 20, 31). Treatment regimens mimicking those utilized in the spectrophotometric analysis were administered following a 24 h biofilm maturation phase on borosilicate glass beads. For strain PAO1 (Fig. 5A), a one-way ANOVA demonstrated a significant treatment effect on bacterial inocula within biofilm (*F*(9, 28) = 6.94, *P* < 0.001, ω = 0.58). Tukey’s HSD *post hoc* tests indicated 109 monotherapy had lower bacterial eradication activity than EM monotherapy (−1.01 vs −2.59 Δlog_10_ CFU/mL, *P* = 0.001) and EC monotherapy (−1.01 vs −2.00 Δlog_10_ CFU/mL, *P* = 0.12). Conversely, when combined with CIP, 109 displayed improved bacterial eradication compared with EC + CIP (−2.42 vs −1.15 Δlog_10_ CFU/mL, *P* = 0.02) and EM + CIP (−2.42 vs −1.87 Δlog_10_ CFU/mL, *P* = 0.55); however, the latter was not statistically significant. The triple-phage cocktail of EM, EC, and 109 did not appear to improve eradication regardless of the addition of CIP against PAO1. Furthermore, the triple-phage cocktail + CIP performed worse than 109 + CIP in bTKAs (−2.42 vs −1.15 Δlog_10_ CFU/mL, *P* = 0.02), which may suggest antagonism between the three phages in this case. No difference was seen between these treatments in the spectrophotometric analysis (mean difference 0.10 ∆OD_600_, *P* = 0.21)

With regard to PA14 (Fig. 5B), one-way ANOVA also confirmed a significant treatment effect on bacterial inocula within biofilm (*F*(9, 30) = 20.39, *P* < 0.001, ω = 0.81). Tukey’s HSD revealed that although 109 appeared to demonstrate greater efficacy as monotherapy (−1.43 Δlog_10_ CFU/mL) compared to EM and EC (−0.70 and −0.52 Δlog_10_ CFU/mL, respectively), these differences were not statistically significant. When combined with CIP, phage EM displayed lower bacterial eradication activity compared to EC + CIP (−0.78 vs −1.64 Δlog_10_ CFU/mL, *P* = 0.11) and 109 + CIP (−0.78 vs −2.14 Δlog_10_ CFU/mL, *P* = 0.001). Notably, there was no statistical difference between CIP monotherapy and combination therapy with any one of the three phages. Interestingly, the triple-phage cocktail did not enhance bacterial eradication relative to EM and EC monotherapy regimens and was inferior to 109 monotherapy (−0.47 vs −1.43, *P* = 0.049). The addition of CIP to the triple-phage cocktail improved eradication compared to the triple-phage cocktail regimen (−0.47 vs −3.18 Δlog_10_ CFU/mL, *P* = 0.004) and CIP monotherapy (−1.73 vs −3.18 Δlog_10_ CFU/mL, *P* = 0.08).

Phages were generally less active against PA14 compared to PAO1, with the exception of 109, which displayed a similar level of activity across both strains. In the absence of CIP, the triple-phage cocktail exhibited decreased efficacy compared to phage monotherapy in several instances across both strains, suggesting the potential for antagonism. However, this potential antagonism did not always manifest in the presence of CIP.

## DISCUSSION

This study assessed the antibiofilm activity of three phages alone and in combination with CIP against *P. aeruginosa* strains producing two distinct biofilm phenotypes. Several notable findings resulted from this study. Firstly, we demonstrated that MIC values were generally increased several-fold in the biofilm state compared to the planktonic state across 12 *P. aeruginosa* strains. This supports previous findings of heightened antibiotic tolerance of bacteria in the biofilm state and underscores the need for additional therapeutic modalities for biofilm-associated infections. We also identified variability in the biofilm production capabilities between our strains; however, the quantity of biofilm produced did not appear to substantially impact MBICs. Strains I0003-1, G0002-2, and AR351, which produced relatively low amounts of biofilm, exhibited MBIC increases of a similar magnitude to those of high biofilm-producing strains. This finding suggests that elements beyond total biomass, such as the composition of the biofilm matrix or bacterial metabolic dormancy, may contribute to antibiotic tolerance.

Additionally, we phenotypically categorized and quantified the relative amounts of exopolysaccharides Psl and Pel present within the biofilms of PAO1 and PA14. As expected, these strains produced distinct biofilms, with PAO1 exhibiting a Psl-rich matrix and PA14 displaying a predominantly Pel-based biofilm. These compositional differences may partially explain the differences in phage susceptibility that we subsequently observed in the biofilm state.

Phage activity differed between the planktonic and biofilm states. In plaque assays, where bacteria are in the planktonic state (albeit spatially restricted by agar), phage EM was the sole highly active phage against PA14 in the planktonic state, whereas it displayed low activity against PAO1. Conversely, phages EC, 109, and EA displayed high activity against PAO1, despite having no activity against PA14. In the spectrophotometric microplate analyses, the data represent overall phage activity against an undefined combination of both planktonic and biofilm CFU, since it measures the transmittance of light through the mixture of biofilm cells adhering to the bottom of the microplate well and suspended cells released from the biofilm. In these assays, phage 109 exhibited generally high activity against both strains in time-kill analyses despite having little activity against PA14 in its planktonic state. This is consistent with previous data suggesting that phage 109 can improve the antibiofilm activity of a cocktail and reduce the development of phage resistance, even if its individual activity is modest (6, 26). It may also suggest that biofilm-specific factors, such as extracellular matrix disruption, may influence phage activity independently of planktonic susceptibility. The addition of CIP greatly enhanced the activity of phages EC and 109 against PA14, with these combinations markedly outperforming either component alone (Fig. 4; Fig. S1).

However, the results of spectrophotometric assays did not always align with the results of the bTKAs. For example, the addition of CIP generally improved bacterial killing in both the spectrophotometric analyses and bTKAs, particularly against PA14, but results with PAO1 exhibited greater variability. Whereas the addition of CIP to phage(s) was neutral or beneficial in the spectrophotometric assays, the addition of CIP to EC against PAO1 biofilms in bTKAs resulted in decreased activity compared to their individual components. Furthermore, the inclusion of EC appeared to decrease the efficacy of the triple-phage cocktail in both PAO1 and PA14 when compared to individual phage performance. This may indicate the possible presence of an antagonistic mechanism involving phage EC. To investigate these findings further, we evaluated the antibiofilm activity of a two-phage cocktail comprising EM and 109 (excluding EC) and compared its activity to that of the triple-phage cocktail of EM, EC, and 109 (Fig. S1). We found that the exclusion of phage EC from the triple-phage cocktail improved antibiofilm activity against strain PA14 (Fig. S1B), but not PAO1 (Fig. S1A). PA14 biofilm regrowth began to appear around 20 h following triple-phage cocktail administration, which may suggest the emergence of resistance to phage. To further explore this observation, we assessed PA14 biofilm proliferation spectrophotometrically on a per-experiment basis. Two of the three biofilm experiments treated with the triple-phage cocktail experienced regrowth between 18 and 24 h after treatment initiation (Fig. S2B), whereas biofilms that were exposed to the two-phage cocktail of EM + 109 did not experience regrowth in any of the three experiments (Fig. S2A). These exploratory data suggest that the addition of EC to phages EM and 109 could facilitate the development of resistance of PA14 to phage. The addition of CIP appeared to minimize this effect. Although excluding EC from the triple-phage cocktail led to marginally improved activity, biofilm eradication was similar between the two-phage and three-phage cocktails (Fig. S1D). It is notable that phages EC and 109 are both members of the *Pbunavirus* genus. While not always the case for phages with similar genomic makeup (32), it is possible that phages EC and 109 compete for the same bacterial receptor, which has the potential to diminish the efficacy of phage cocktails and accelerate the selection of phage-resistant mutants (33). Several *Pbunavirus* phages have been shown to bind LPS (34, 35), but there is also evidence that they may use multiple components of LPS or other receptor(s) in addition to LPS (36). Therefore, additional work to delineate how EC and 109 use specific cell surface receptors could help to explain our observations. Notably, previous data suggest that the addition of 109 to EM + EC, with or without CIP, prevented the development of resistance to EM and EC in *P. aeruginosa* strain AR351 (6). These contrasting results indicate that bacterial phage-resistance development may be strain-specific, and the impact of biofilm phenotype on this phenomenon remains of interest in future studies. This type of strain specificity has been previously reported (37). In *Staphylococcus aureus*, we have tentatively identified a combination of phages and antibiotics that seems to substantially reduce this type of strain specificity (20, 21, 38), but without further understanding the underlying mechanisms, it is not clear whether that observation will be generalizable. Future studies should aim to elucidate the mechanisms by which EC might be antagonistic within the triple-phage cocktail of EM, EC, and 109.

It would also be of interest to identify and characterize the enzymatic specificities of these phages’ tailspike proteins. The best-studied phage depolymerases that are classically associated with enhanced biofilm degradation are encoded by podophages and generate expanding, diffusible halos in plaque assays due to excess free tailspike depolymerase that is released from lysed cells. More recently, the enzymatic activities of myophage and siphophage tailspike proteins have begun to be better characterized (24). Phages EM, EC, and 109 are myophages that do not produce expanding plaque halos, although we do observe that EM plaques have a clear center surrounded by a turbid zone that could indicate a less readily diffusible depolymerase activity (Fig. S3). However, the specific activities of non-podophage tailspikes are difficult to predict from sequence data. For example, the most promising depolymerase candidate predicted by PhageDPO, a trained Support Vector Machine model for predicting depolymerases (39), was high confidence (≥90%) for phage EC (locus EC_069, 96%), slightly lower confidence for phage 109 (locus 109_069, 88%), and not confident for EM (locus EM_097, 73%). However, while all of these genes cluster with other tail components in the phage genomes, none of the proteins have domains that were recognized as lyases, hydrolases, or sialidases by CDD-Search or Phyre2. Either these proteins are not depolymerases, or they are too different from characterized depolymerases to be easily identified. In the future, this could be tested by purifying these and other probable tail proteins in order to observe their activity directly.

Our study highlights the potential impact of biofilm composition on phage activity in *P. aeruginosa*. While we were able to characterize differences in phage activity between Class I and Class II biofilms, it is unknown if these findings will extend to other strains and biofilm phenotypes, such as Class III. Another limitation of our study was the disparity in biofilm maturity between the exopolysaccharide quantification assay and the phage activity experiments (spectrophotometry and bTKAs). While an extended growth phase (up to 96 h) is often necessary to obtain high-quality images of biofilms within flow cells (9), it is possible that the exopolysaccharide content of *P. aeruginosa* biofilms may vary with maturity. This, in turn, could alter how phages are able to bind to biofilm cells and disseminate within the biofilm. Future directions will involve phenotyping the biofilms of clinical *P. aeruginosa* isolates to further establish the relationship between phage activity and biofilm composition, with the aim of developing a phage-antibiotic cocktail with the potential for activity across a diverse array of biofilm phenotypes without the need for strain-specific phenotype identification.

## MATERIALS AND METHODS

### Bacterial strains and bacteriophages

The panel of *P. aeruginosa* strains in this study consisted of 10 well-characterized clinical isolates (AR351, AR352, AR353, AR356, AR357, HFHS22, I0003-1, G0002-2, K0001-1, K0003-1), along with two laboratory standard strains (PAO1 and PA14). Of the 10 clinical isolates, 4 were phenotypically classified as MDR, and 6 were classified as XDR. Laboratory standard strains PAO1 and PA14 were purchased from the American Type Culture Collection. Isolates K0001-1, G0002-2, K0003-1, and I0003-1 from the OVERCOME study were kindly provided by Dr. Keith Kaye (40). Strains AR351, AR352, AR353, AR356, and AR357 were obtained from the US Centers for Disease Control and Prevention AR Isolate Bank. HFHS22 was isolated from a patient hospitalized at Henry Ford Hospital in Detroit, Michigan. All bacterial strains have been sequenced and are available at the National Center for Biotechnology Information (BioProject PRJNA939146). Phages EM-T3762627-2_AH (EM) and LL-5504721-AH (LL) were provided by Dr. Jose Alexander (AdventHealth Orlando, Winter Springs, FL, USA). Phages 109, Paer4, E2005-C (EC), and E2005-A (EA) were provided by Dr. Rodney Donlan (Centers for Disease Control and Prevention, Atlanta, GA, USA) and have been previously described (41); phage 109 was originally part of a typing set described by Lindberg and Latta (42). All phages have been sequenced and speciated (Fig. 1). The genomes of the three main phages used in this work are available in GenBank: OQ831730.1 (109), OQ831729.1 (E2005-C), ON169972.1 (EM). The genomes of phages EA, Paer4, and LL are also available in GenBank: PX277137 (EA), PX277136 (Paer4), PX277138 (LL). The complete predicted proteome of each phage was processed using CDD-Search (43, 44) with the default parameters to identify conserved domains. Depolymerase predictions for EC, 109, and EM were made using PhageDPO, as available through the Galaxy toolshed (39), and the best candidate from each phage (all by a >20-point margin) was additionally run through Phyre2 (45) with the default parameters to identify specific enzymatic domains.

### MIC/MBIC determination

MICs for each strain were determined in duplicate by performing microbroth dilution at ~5 × 10^5^ CFU/mL with cation-adjusted Mueller-Hinton broth (CAMHB) (iron-depleted CAMHB for cefiderocol) in accordance with the Clinical and Laboratory Standards Institute M100 Performance Standards for Antimicrobial Susceptibility Testing (34th Edition) handbook (46). Anti-pseudomonal antibiotics evaluated comprised meropenem, colistin, amikacin, aztreonam, CIP, ceftolozane-tazobactam, and cefiderocol. MBICs were determined utilizing the pin-lid method (Calgary Biofilm Device) as previously described (28). Briefly, 150 µL of a bacterial stock solution made with ~1 × 10^7^ CFU/mL bacteria of interest suspended in 10 mL of glucose-supplemented tryptic soy broth (GSTSB) (iron-depleted CAMHB for cefiderocol) was added to all wells of a polystyrene 96-well plate, reserving one row for media control. A transferable solid-phase pin lid was placed onto the microtiter plate and was incubated for 24 h at 37°C. Following incubation, the pin lid was removed, rinsed with sterilized phosphate-buffered saline to remove planktonic bacteria, and placed onto a new 96-well plate with serially diluted drugs of interest in GSTSB. The microtiter plate was incubated for 24 h at 37°C, after which the pin lid was removed and the MBICs were determined visually.

### Planktonic phage activity

The activity of phages against *P. aeruginosa* strains of interest was determined via agar plaque assay using 10-fold serial dilutions of phage suspension. Briefly, phages were propagated via 24 h incubation with liquid culture of the host organism in brain heart infusion (BHI) broth. Following incubation, the lysate was centrifuged at 4,000 rpm to pellet the remaining bacterial debris. The supernatant was withdrawn and purified using a 0.22 µm filter. Phage activity was quantified via agar plaque assay as follows: a bacterial lawn was created using a 4.0 McFarland suspension of bacteria of interest, which was mixed with molten brain heart infusion 0.7% agar overlay, then poured onto BHI agar plates and allowed to set. Phages were serially diluted (1:10) using phosphate-buffered saline in a polystyrene 96-well plate, then plated onto the inoculated agar overlay and incubated for 24 h at 37°C. Following incubation, phage plaques were counted, allowing for the calculation of the titer in plaque-forming units per milliliter. This process was first conducted against each phage’s host to assess the degree of propagation, then diluted to create a 1 × 10^7^ PFU/mL phage stock. This procedure was subsequently performed against each bacterial target strain to determine phage activity. Phages were considered to have high activity against the bacterial target strain if titers exceeded 1 × 10^7^ PFU/mL, low activity if titers were between 1 × 10^3^ and 1 × 10^7^ PFU/mL, and no activity if titers were less than 1 × 10^3^ PFU/mL.

### Biofilm quantification

The biofilm of each strain of interest was quantified using spectrophotometry at OD_570_ utilizing methods previously described (47). Briefly, 50 µL GSTSB was inoculated with the bacterial strain of interest to achieve a final inoculum of ~5 × 10^5^ CFU/mL, placed into wells of a flat-bottom polystyrene 96-well plate, and incubated for 24 h at 37°C. After 24 h of incubation, the growth media was removed, and each well was rinsed three times with 0.9% NaCl to wash out remaining planktonic bacteria. The remaining biofilm present within each well was fixed with methanol, then stained for 5 minutes with a 2% crystal violet solution. Excess stain was rinsed away using deionized water. The plate was allowed to dry for 1 h in a sterile biosafety cabinet. Following the drying period, the dye was resolubilized and extracted using 33% glacial acetic acid and transferred to a fresh plate to read the optical density at 570 nm. Each experiment was performed in triplicate.

### Biofilm exopolysaccharide quantification

Exopolysaccharides Pel and Psl present within the biofilms of non-mucoid *P. aeruginosa* were imaged in the biofilms of strains PAO1 and PA14 to assess their relative quantities. Biofilms were grown within aluminum transmission continuous flow cells (BioSurface Technologies, Bozeman, MT, USA) harboring a glass microscope slide and coverslip. A 0.5 McFarland suspension of the bacterial strain of interest was injected into the flow cell and was allowed to adhere to the glass slide for 1 h. Subsequently, 1% GSTSB was continuously supplied to the flow cell at a rate of 10 mL/h. Biofilms were developed for 96 h at 37°C. Following this growth phase, biofilm exopolysaccharides were labeled with specific fluorophores for Psl (50 µg/mL TRITC-conjugated Hippeastrum hybrid lectin, EY Laboratories, San Mateo, CA, USA), Pel (100 µg/mL fluorescein-conjugated Wisteria floribunda lectin, Vector Laboratories, Newark, CA, USA), and general biomass (5 µM Syto62, Invitrogen, Waltham, Massachusetts, USA) using a technique adapted from Jennings et al. (9). Flow cells were then disassembled, and microscope slides were fixed with an antifade mountant (Invitrogen, Waltham, MA, USA). Biofilm slides were imaged at the Microscopy, Imaging, and Cytometry Resources Core at Wayne State University School of Medicine, using a Zeiss LSM 780 laser scanning confocal microscope (Carl Zeiss AG, Oberkochen, Baden-Württemberg, Germany). Images were processed using Zeiss ZEN and ImageJ software. Fluorescent intensity normalization was performed for visualization purposes. Pixel analysis was conducted using ImageJ software to determine the relative quantities of each exopolysaccharide relative to general biomass. Briefly, raw images were filtered using a median filter with a radius of 2 pixels to reduce noise. A binary threshold mask was applied to each fluorescence channel to exclude background signal and isolate regions of interest for quantification. The number of pixels above the threshold in each channel was quantified and represented as a fraction of the total biomass pixel area.

### Spectrophotometric analysis of phage-antibiotic activity against established biofilms

Biofilm growth analysis was performed using spectrophotometry assessing changes in optical density at 600 nm (OD_600_) utilizing a LogPhase 600 microplate spectrophotometer (Agilent, Santa Clara, CA, USA). Briefly, 200 µL of GSTSB was inoculated with the bacterial strain of interest to achieve a final inoculum of ~5 × 10^5^ CFU/mL and added to the wells of a tissue culture-treated 96-well flat-bottom plate. For the biofilm growth phase, inoculated microtiter plates were sealed with a gas-permeable film (USA Scientific, Ocala, FL, USA) and placed within the spectrophotometer, where they were incubated at 37°C and rotated at 500 rpm for 24 h. OD_600_ was analyzed every 10 minutes to monitor the biofilm growth phase. After 24 h, the plates were removed from the spectrophotometer, and the media in each well was discarded. Each well was rinsed twice with 0.9% NaCl to remove any remaining planktonic bacteria. New media, along with the designated treatments (phage[s] ± CIP), were added to each well. Phages were propagated in brain heart infusion broth and dosed at MOI ≅ 1, and CIP was dosed at 0.5× MIC to reflect the subinhibitory concentrations often observed within the biofilm matrix due to limited antimicrobial penetration (6, 48). An MOI of 1.0 was selected based on the results of previously published phage dose optimization experiments involving these phages and bacterial strains (49). Microtiter plates were again sealed with a gas-permeable film and placed in the spectrophotometer at 37°C and rotated at 500 rpm for 24 h. OD_600_ was read every 10 minutes until 24 h elapsed. All experiments were conducted in triplicate. Bacterial density curves (Fig. 4) were generated using the difference in OD_600_ readings compared to growth control after normalization to the initial (T_0_) readings. Differences between treatments were assessed using a one-way ANOVA, followed by Tukey’s HSD *post hoc* test for multiple comparisons (equal variances confirmed by the Brown-Forsythe test).

### Biofilm time-kill analyses

Time-kill analyses were conducted for assessment of antibiofilm activity using methods previously described (49). Briefly, borosilicate glass beads were placed within the wells of a non-tissue culture-treated 24-well plate with 2 mL of GSTSB added to each well. Each well was subsequently inoculated with one colony (~1 µL) of target bacteria. Plates were incubated at 37°C at 50 rpm for 24 h to allow for biofilm formation. Following this biofilm growth phase, glass beads (now coated with biofilm) were extracted from each well and placed in a new 24-well plate with fresh GSTSB, along with the designated treatment modalities (phage[s] ± CIP) and incubated at 37°C at 50 rpm for 24 h. Phages were propagated in brain heart infusion broth and dosed at MOI ≅ 1, and CIP was dosed at 0.5× MIC to reflect the subinhibitory concentrations often observed within the biofilm matrix (6, 48). For sampling, beads were removed, processed via sonication, then plated for total bacterial counts at hours 0 and 24. All experiments were completed in duplicate. Differences in CFU/mL between the initial and final samples were plotted in Fig. 5. Similarly to the spectrophotometric analyses, differences between treatments were assessed using a one-way ANOVA, followed by Tukey’s HSD *post hoc* test for multiple comparisons (equal variances confirmed by the Brown-Forsythe test).

## SUPPLEMENTAL MATERIAL

Supplemental material aac.00925-25-s0001.docx Fig. S1 to S3; Tables S1 and S2.

ASM does not own the copyrights to Supplemental Material that may be linked to, or accessed through, an article. The authors have granted ASM a non-exclusive, world-wide license to publish the Supplemental Material files. Please contact the corresponding author directly for reuse.

## References

[1] Scott RD. 2009. The direct medical costs of healthcare-associated infections in U.S. hospitals and the benefits of prevention CDC

[2] Horcajada JP, Montero M, Oliver A, Sorlí L, Luque S, Gómez-Zorrilla S, Benito N, Grau S. 2019. Epidemiology and treatment of multidrug-resistant and extensively drug-resistant Pseudomonas aeruginosa infections. Clin Microbiol Rev 32:e00031-19. doi:10.1128/CMR.00031-19 31462403 PMC6730496

[3] Singh S, Singh SK, Chowdhury I, Singh R. 2017. Understanding the mechanism of bacterial biofilms resistance to antimicrobial agents. Open Microbiol J 11:53–62. doi:10.2174/1874285801711010053 28553416 PMC5427689

[4] Schaible B, Taylor CT, Schaffer K. 2012. Hypoxia increases antibiotic resistance in Pseudomonas aeruginosa through altering the composition of multidrug efflux pumps. Antimicrob Agents Chemother 56:2114–2118. doi:10.1128/AAC.05574-11 22290986 PMC3318321

[5] Bagge N, Hentzer M, Andersen JB, Ciofu O, Givskov M, Høiby N. 2004. Dynamics and spatial distribution of beta-lactamase expression in Pseudomonas aeruginosa biofilms. Antimicrob Agents Chemother 48:1168–1174. doi:10.1128/AAC.48.4.1168-1174.2004 15047517 PMC375278

[6] Holger DJ, El Ghali A, Bhutani N, Lev KL, Stamper K, Kebriaei R, Kunz Coyne AJ, Morrisette T, Shah R, Alexander J, Lehman SM, Rojas LJ, Marshall SH, Bonomo RA, Rybak MJ. 2023. Phage-antibiotic combinations against multidrug-resistant Pseudomonas aeruginosa in in vitro static and dynamic biofilm models. Antimicrob Agents Chemother 67:e0057823. doi:10.1128/aac.00578-23 37855639 PMC10648846

[7] Moradali MF, Ghods S, Rehm BHA. 2017. Pseudomonas aeruginosa lifestyle: a paradigm for adaptation, survival, and persistence. Front Cell Infect Microbiol 7:39. doi:10.3389/fcimb.2017.00039 28261568 PMC5310132

[8] Byrd MS, Sadovskaya I, Vinogradov E, Lu H, Sprinkle AB, Richardson SH, Ma L, Ralston B, Parsek MR, Anderson EM, Lam JS, Wozniak DJ. 2009. Genetic and biochemical analyses of the Pseudomonas aeruginosa Psl exopolysaccharide reveal overlapping roles for polysaccharide synthesis enzymes in Psl and LPS production. Mol Microbiol 73:622–638. doi:10.1111/j.1365-2958.2009.06795.x 19659934 PMC4409829

[9] Jennings LK, Storek KM, Ledvina HE, Coulon C, Marmont LS, Sadovskaya I, Secor PR, Tseng BS, Scian M, Filloux A, Wozniak DJ, Howell PL, Parsek MR. 2015. Pel is a cationic exopolysaccharide that cross-links extracellular DNA in the Pseudomonas aeruginosa biofilm matrix. Proc Natl Acad Sci USA 112:11353–11358. doi:10.1073/pnas.1503058112 26311845 PMC4568648

[10] Colvin KM, Irie Y, Tart CS, Urbano R, Whitney JC, Ryder C, Howell PL, Wozniak DJ, Parsek MR. 2012. The Pel and Psl polysaccharides provide Pseudomonas aeruginosa structural redundancy within the biofilm matrix. Environ Microbiol 14:1913–1928. doi:10.1111/j.1462-2920.2011.02657.x 22176658 PMC3840794

[11] Ghafoor A, Hay ID, Rehm BHA. 2011. Role of exopolysaccharides in Pseudomonas aeruginosa biofilm formation and architecture. Appl Environ Microbiol 77:5238–5246. doi:10.1128/AEM.00637-11 21666010 PMC3147449

[12] Colvin KM, Gordon VD, Murakami K, Borlee BR, Wozniak DJ, Wong GCL, Parsek MR. 2011. The pel polysaccharide can serve a structural and protective role in the biofilm matrix of Pseudomonas aeruginosa. PLoS Pathog 7:e1001264. doi:10.1371/journal.ppat.1001264 21298031 PMC3029257

[13] Holger D, Kebriaei R, Morrisette T, Lev K, Alexander J, Rybak M. 2021. Clinical pharmacology of bacteriophage therapy: a focus on multidrug-resistant Pseudomonas aeruginosa infections. Antibiotics (Basel) 10:556. doi:10.3390/antibiotics10050556 34064648 PMC8151982

[14] Wright A, Hawkins CH, Anggård EE, Harper DR. 2009. A controlled clinical trial of a therapeutic bacteriophage preparation in chronic otitis due to antibiotic-resistant Pseudomonas aeruginosa; a preliminary report of efficacy. Clin Otolaryngol 34:349–357. doi:10.1111/j.1749-4486.2009.01973.x 19673983

[15] Ooi ML, Drilling AJ, Morales S, Fong S, Moraitis S, Macias-Valle L, Vreugde S, Psaltis AJ, Wormald PJ. 2019. Safety and tolerability of bacteriophage therapy for chronic rhinosinusitis due to Staphylococcus aureus. JAMA Otolaryngol Head Neck Surg 145:723–729. doi:10.1001/jamaoto.2019.1191 31219531 PMC6587246

[16] Kebriaei R, Lehman SM, Shah RM, Stamper KC, Kunz Coyne AJ, Holger D, El Ghali A, Rybak MJ. 2023. Optimization of phage-antibiotic combinations against Staphylococcus aureus biofilms. Microbiol Spectr 11:e0491822. doi:10.1128/spectrum.04918-22 37199616 PMC10269792

[17] El Ghali A, Stamper K, Kunz Coyne AJ, Holger D, Kebriaei R, Alexander J, Lehman SM, Rybak MJ. 2023. Ciprofloxacin in combination with bacteriophage cocktails against multi-drug resistant Pseudomonas aeruginosa in ex vivo simulated endocardial vegetation models. Antimicrob Agents Chemother 67:e0072823. doi:10.1128/aac.00728-23 37877697 PMC10649104

[18] Shariati A, Noei M, Chegini Z. 2023. Bacteriophages: the promising therapeutic approach for enhancing ciprofloxacin efficacy against bacterial infection. J Clin Lab Anal 37:e24932. doi:10.1002/jcla.24932 37377167 PMC10388223

[19] Ferran AA, Lacroix MZ, Gourbeyre O, Huesca A, Gaborieau B, Debarbieux L, Bousquet-Mélou A. 2022. The selection of antibiotic- and bacteriophage-resistant pseudomonas aeruginosa is prevented by their combination. Microbiol Spectr 10:e0287422. doi:10.1128/spectrum.02874-22 36135376 PMC9602269

[20] Kunz Coyne AJ, Stamper K, Bleick C, Kebriaei R, Lehman SM, Rybak MJ. 2024. Synergistic bactericidal effects of phage-enhanced antibiotic therapy against MRSA biofilms. Microbiol Spectr 12:e0321223. doi:10.1128/spectrum.03212-23 38411110 PMC10986480

[21] Kunz Coyne AJ, Bleick C, Stamper K, Kebriaei R, Bayer AS, Lehman SM, Rybak MJ. 2024. Phage-antibiotic synergy against daptomycin-nonsusceptible MRSA in an ex vivo simulated endocardial pharmacokinetic/pharmacodynamic model. Antimicrob Agents Chemother 68:e0138823. doi:10.1128/aac.01388-23 38376187 PMC10989002

[22] Kunz Coyne AJ, Eshaya M, Bleick C, Vader S, Biswas B, Wilson M, Deschenes MV, Alexander J, Lehman SM, Rybak MJ. 2024. Exploring synergistic and antagonistic interactions in phage-antibiotic combinations against ESKAPE pathogens. Microbiol Spectr. doi:10.1128/spectrum.00427-24:e0042724 PMC11468199 39082827

[23] Kunz Coyne AJ, Stamper K, Kebriaei R, Holger DJ, El Ghali A, Morrisette T, Biswas B, Wilson M, Deschenes MV, Canfield GS, Duerkop BA, Arias CA, Rybak MJ. 2022. Phage cocktails with daptomycin and ampicillin eradicates biofilm-embedded multidrug-resistant Enterococcus faecium with preserved phage susceptibility. Antibiotics (Basel) 11:1175. doi:10.3390/antibiotics11091175 36139953 PMC9495159

[24] Knecht LE, Veljkovic M, Fieseler L. 2019. Diversity and function of phage encoded depolymerases. Front Microbiol 10:2949. doi:10.3389/fmicb.2019.02949 31998258 PMC6966330

[25] O’Flynn G, Ross RP, Fitzgerald GF, Coffey A. 2004. Evaluation of a cocktail of three bacteriophages for biocontrol of Escherichia coli O157:H7. Appl Environ Microbiol 70:3417–3424. doi:10.1128/AEM.70.6.3417-3424.2004 15184139 PMC427753

[26] Lehman SM, Donlan RM. 2015. Bacteriophage-mediated control of a two-species biofilm formed by microorganisms causing catheter-associated urinary tract infections in an in vitro urinary catheter model. Antimicrob Agents Chemother 59:1127–1137. doi:10.1128/AAC.03786-14 25487795 PMC4335898

[27] Billaud M, Plantady C, Lerouge B, Ollivier E, Lossouarn J, Moncaut E, Deschamps J, Briandet R, Cleret A, Fevre C, Demarre G, Petit MA. 2025. Complementary killing activities of pbunavirus LS1 and bruynoghevirus LUZ24 phages on planktonic and sessile Pseudomonas aeruginosa PAO1 derivatives. Antimicrob Agents Chemother. doi:10.1128/aac.00579-25:e0057925 PMC12406683 40698811

[28] Ceri H, Olson ME, Stremick C, Read RR, Morck D, Buret A. 1999. The calgary biofilm device: new technology for rapid determination of antibiotic susceptibilities of bacterial biofilms. J Clin Microbiol 37:1771–1776. doi:10.1128/JCM.37.6.1771-1776.1999 10325322 PMC84946

[29] Heydorn A, Ersbøll B, Kato J, Hentzer M, Parsek MR, Tolker-Nielsen T, Givskov M, Molin S. 2002. Statistical analysis of Pseudomonas aeruginosa biofilm development: impact of mutations in genes involved in twitching motility, cell-to-cell signaling, and stationary-phase sigma factor expression. Appl Environ Microbiol 68:2008–2017. doi:10.1128/AEM.68.4.2008-2017.2002 11916724 PMC123874

[30] Chaudhry WN, Concepción-Acevedo J, Park T, Andleeb S, Bull JJ, Levin BR. 2017. Synergy and order effects of antibiotics and phages in killing Pseudomonas aeruginosa biofilms. PLoS One 12:e0168615. doi:10.1371/journal.pone.0168615 28076361 PMC5226664

[31] Lev K, Kunz Coyne AJ, Kebriaei R, Morrisette T, Stamper K, Holger DJ, Canfield GS, Duerkop BA, Arias CA, Rybak MJ. 2022. Evaluation of bacteriophage-antibiotic combination therapy for biofilm-embedded MDR Enterococcus faecium. Antibiotics (Basel) 11:392. doi:10.3390/antibiotics11030392 35326855 PMC8944492

[32] Ma R, Lai J, Chen X, Wang L, Yang Y, Wei S, Jiao N, Zhang R. 2021. A novel phage infecting Alteromonas represents a distinct group of siphophages infecting diverse aquatic copiotrophs. mSphere 6:e0045421. doi:10.1128/mSphere.00454-21 34106770 PMC8265664

[33] Bürkle M, Korf IHE, Lippegaus A, Krautwurst S, Rohde C, Weissfuss C, Nouailles G, Tene XM, Gaborieau B, Ghigo J-M, Ricard J-D, Hocke AC, Papenfort K, Debarbieux L, Witzenrath M, Wienhold S-M, Krishnamoorthy G. 2025. Phage-phage competition and biofilms affect interactions between two virulent bacteriophages and Pseudomonas aeruginosa. ISME J 19:wraf065. doi:10.1093/ismejo/wraf065 40188480 PMC12041424

[34] Jarrell K, Kropinski AM. 1977. Identification of the cell wall receptor for bacteriophage E79 in Pseudomonas aeruginosa strain PAO. J Virol 23:461–466. doi:10.1128/JVI.23.3.461-466.1977 408513 PMC515855

[35] Garbe J, Wesche A, Bunk B, Kazmierczak M, Selezska K, Rohde C, Sikorski J, Rohde M, Jahn D, Schobert M. 2010. Characterization of JG024, a pseudomonas aeruginosa PB1-like broad host range phage under simulated infection conditions. BMC Microbiol 10:301. doi:10.1186/1471-2180-10-301 21110836 PMC3008698

[36] Uchiyama J, Suzuki M, Nishifuji K, Kato SI, Miyata R, Nasukawa T, Yamaguchi K, Takemura-Uchiyama I, Ujihara T, Shimakura H, Murakami H, Okamoto N, Sakaguchi Y, Shibayama K, Sakaguchi M, Matsuzaki S. 2016. Analyses of short-term antagonistic evolution of Pseudomonas aeruginosa strain PAO1 and phage KPP22 (myoviridae family, PB1-like virus genus). Appl Environ Microbiol 82:4482–4491. doi:10.1128/AEM.00090-16 27208109 PMC4984277

[37] Martin I, Morales S, Alton EWFW, Davies JC. 2023. Lytic bacteriophage is a promising adjunct to common antibiotics across cystic fibrosis clinical strains and culture models of Pseudomonas aeruginosa infection. Antibiotics (Basel) 12:593. doi:10.3390/antibiotics12030593 36978460 PMC10044644

[38] Kebriaei R, Lev KL, Stamper KC, Lehman SM, Morales S, Rybak MJ. 2020. Bacteriophage AB-SA01 cocktail in combination with antibiotics against MRSA-VISA strain in an in vitro pharmacokinetic/pharmacodynamic model. Antimicrob Agents Chemother 65:e01863-20. doi:10.1128/AAC.01863-20 33077648 PMC7927802

[39] Vieira MF, Duarte J, Domingues R, Oliveira H, Dias O. 2025. PhageDPO: a machine-learning based computational framework for identifying phage depolymerases. Comput Biol Med 188:109836. doi:10.1016/j.compbiomed.2025.109836 39951981

[40] Kaye KS, Marchaim D, Thamlikitkul V, Carmeli Y, Chiu CH, Daikos G, Dhar S, Durante-Mangoni E, Gikas A, Kotanidou A, et al.. 2023. Colistin monotherapy versus combination therapy for carbapenem-resistant organisms. NEJM Evidence 2. doi:10.1056/EVIDoa2200131 PMC10398788 37538951

[41] Fu W, Forster T, Mayer O, Curtin JJ, Lehman SM, Donlan RM. 2010. Bacteriophage cocktail for the prevention of biofilm formation by Pseudomonas aeruginosa on catheters in an in vitro model system. Antimicrob Agents Chemother 54:397–404. doi:10.1128/AAC.00669-09 19822702 PMC2798481

[42] Lindberg RB, Latta RL. 1974. Phage typing of Pseudomonas aeruginosai clinical and epidemiologic considerations. Journal of Infectious Diseases 130:S33–S42. doi:10.1093/infdis/130.Supplement.S33 4213792

[43] Marchler-Bauer A, Bryant SH. 2004. CD-search: protein domain annotations on the fly. Nucleic Acids Res 32:W327–31. doi:10.1093/nar/gkh454 15215404 PMC441592

[44] Marchler-Bauer A, Lu S, Anderson JB, Chitsaz F, Derbyshire MK, DeWeese-Scott C, Fong JH, Geer LY, Geer RC, Gonzales NR, et al.. 2011. CDD: a conserved domain database for the functional annotation of proteins. Nucleic Acids Res 39:D225–9. doi:10.1093/nar/gkq1189 21109532 PMC3013737

[45] Kelley LA, Mezulis S, Yates CM, Wass MN, Sternberg MJE. 2015. The Phyre2 web portal for protein modeling, prediction and analysis. Nat Protoc 10:845–858. doi:10.1038/nprot.2015.053 25950237 PMC5298202

[46] Anonymous. 2024. CLSI M100-ED34:2024 performance standards for antimicrobial susceptibility testing. 34th ed. Clinical and Laboratory Standards Institute.

[47] Stepanovic S, Vukovic D, Dakic I, Savic B, Svabic-Vlahovic M. 2000. A modified microtiter-plate test for quantification of staphylococcal biofilm formation. J Microbiol Methods 40:175–179. doi:10.1016/s0167-7012(00)00122-6 10699673

[48] Elfadadny A, Ragab RF, AlHarbi M, Badshah F, Ibáñez-Arancibia E, Farag A, Hendawy AO, De Los Ríos-Escalante PR, Aboubakr M, Zakai SA, Nageeb WM. 2024. Antimicrobial resistance of Pseudomonas aeruginosa: navigating clinical impacts, current resistance trends, and innovations in breaking therapies. Front Microbiol 15:1374466. doi:10.3389/fmicb.2024.1374466 38646632 PMC11026690

[49] Holger DJ, Lev KL, Kebriaei R, Morrisette T, Shah R, Alexander J, Lehman SM, Rybak MJ. 2022. Bacteriophage-antibiotic combination therapy for multidrug-resistant Pseudomonas aeruginosa: in vitro synergy testing. J Appl Microbiol 133:1636–1649. doi:10.1111/jam.15647 35652690

