# Supplementary material for: Impact of Pseudomonas aeruginosa biofilm exopolysaccharide composition on bacteriophage and bacteriophage-antibiotic combination activity
Source: Antimicrob Agents Chemother. 2025 Dec 5;70(1):e00925-25. doi: 10.1128/aac.00925-25 (PMC12777554; doi:10.1128/aac.00925-25)
Supplement: Supplemental material — Fig. S1 to S3; Tables S1 and S2. [file aac.00925-25-s0001.docx]

**Impact of Pseudomonas aeruginosa biofilm exopolysaccharide composition on bacteriophage and bacteriophage-antibiotic combination activity.**

**Supplementary Material**

|  | |
| --- | --- |
| 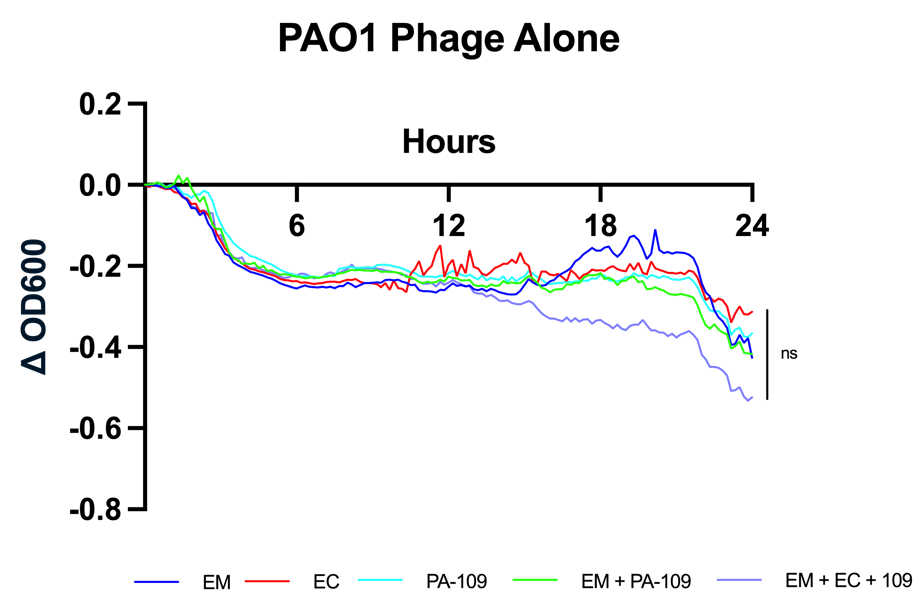  **A. Phage Alone: PAO1** | 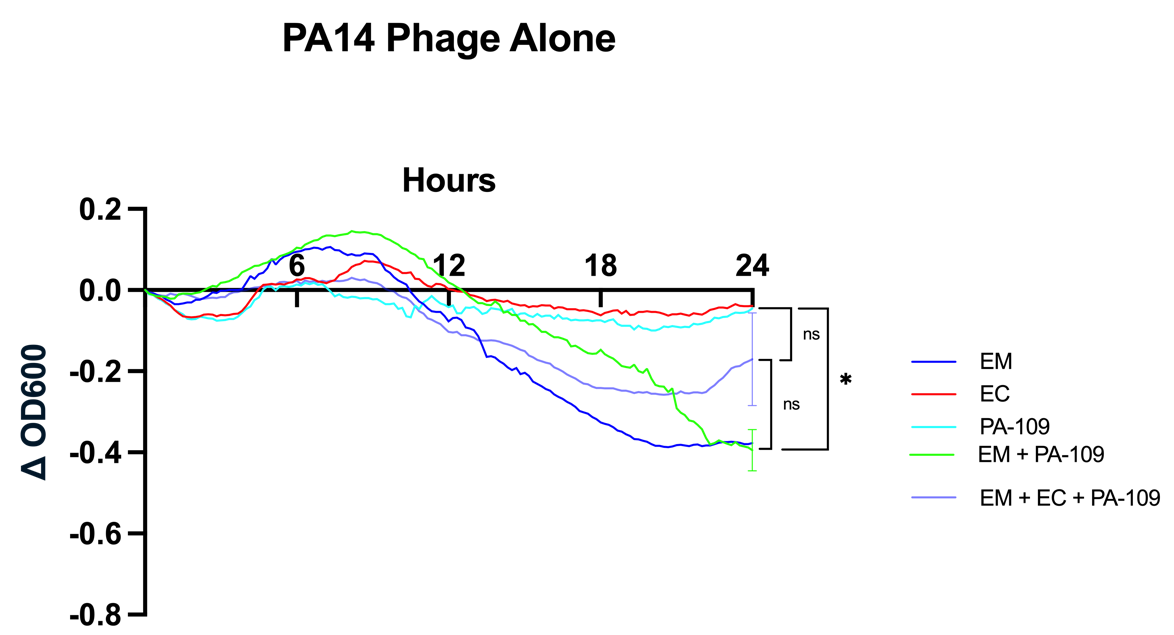  **B. Phage Alone: PA14** |
| 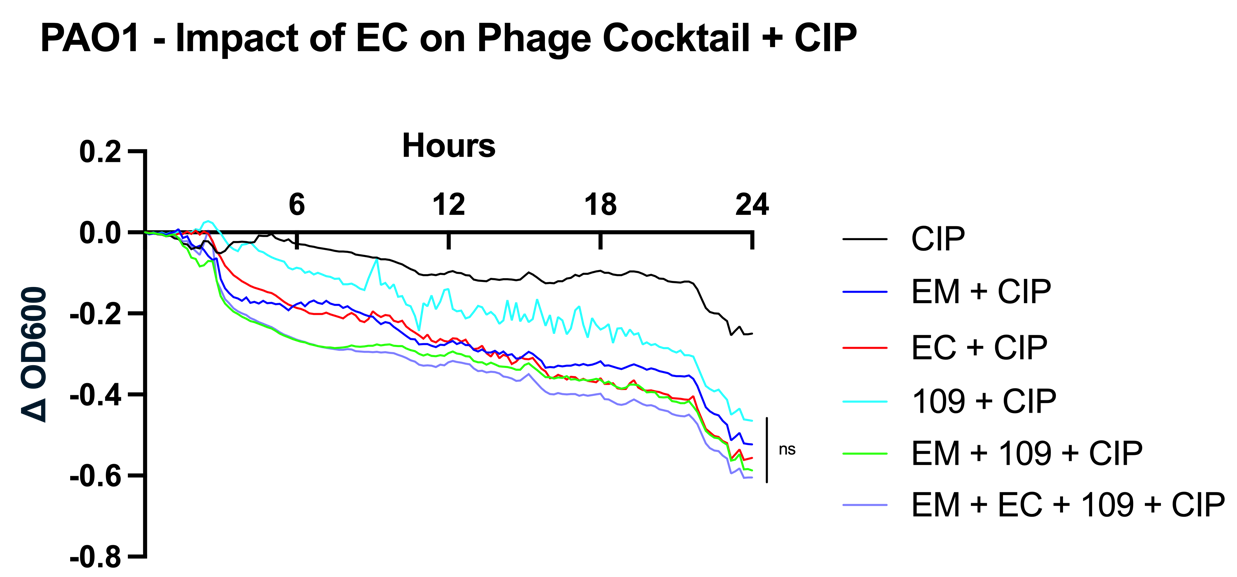  **C. Phage + CIP: PAO1** | 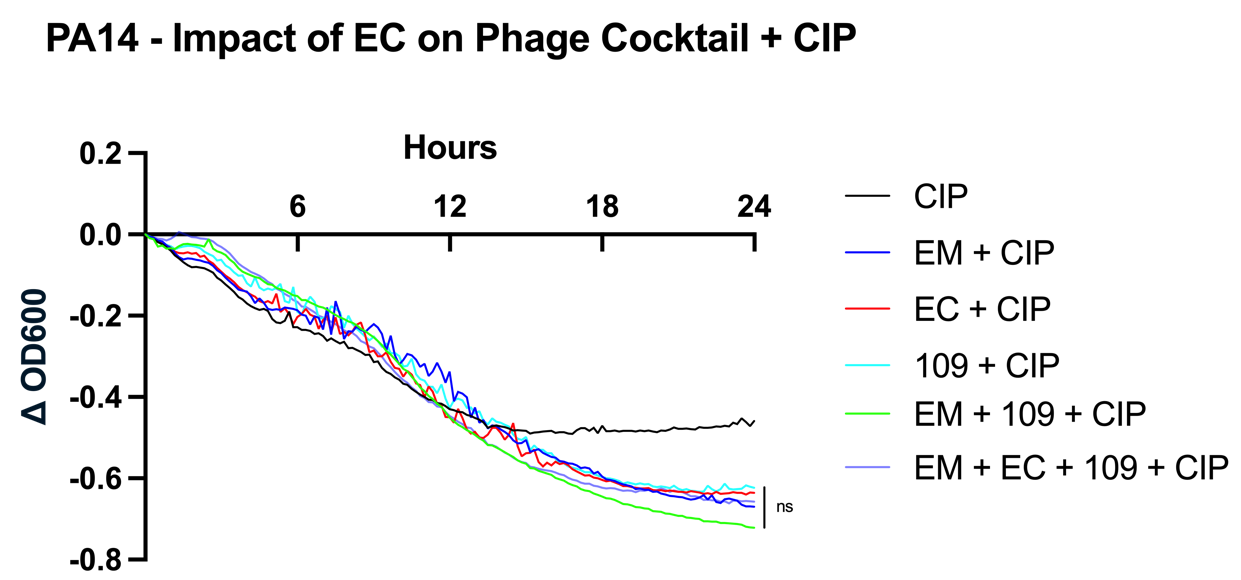  **D. Phage + CIP: PA14** |
| **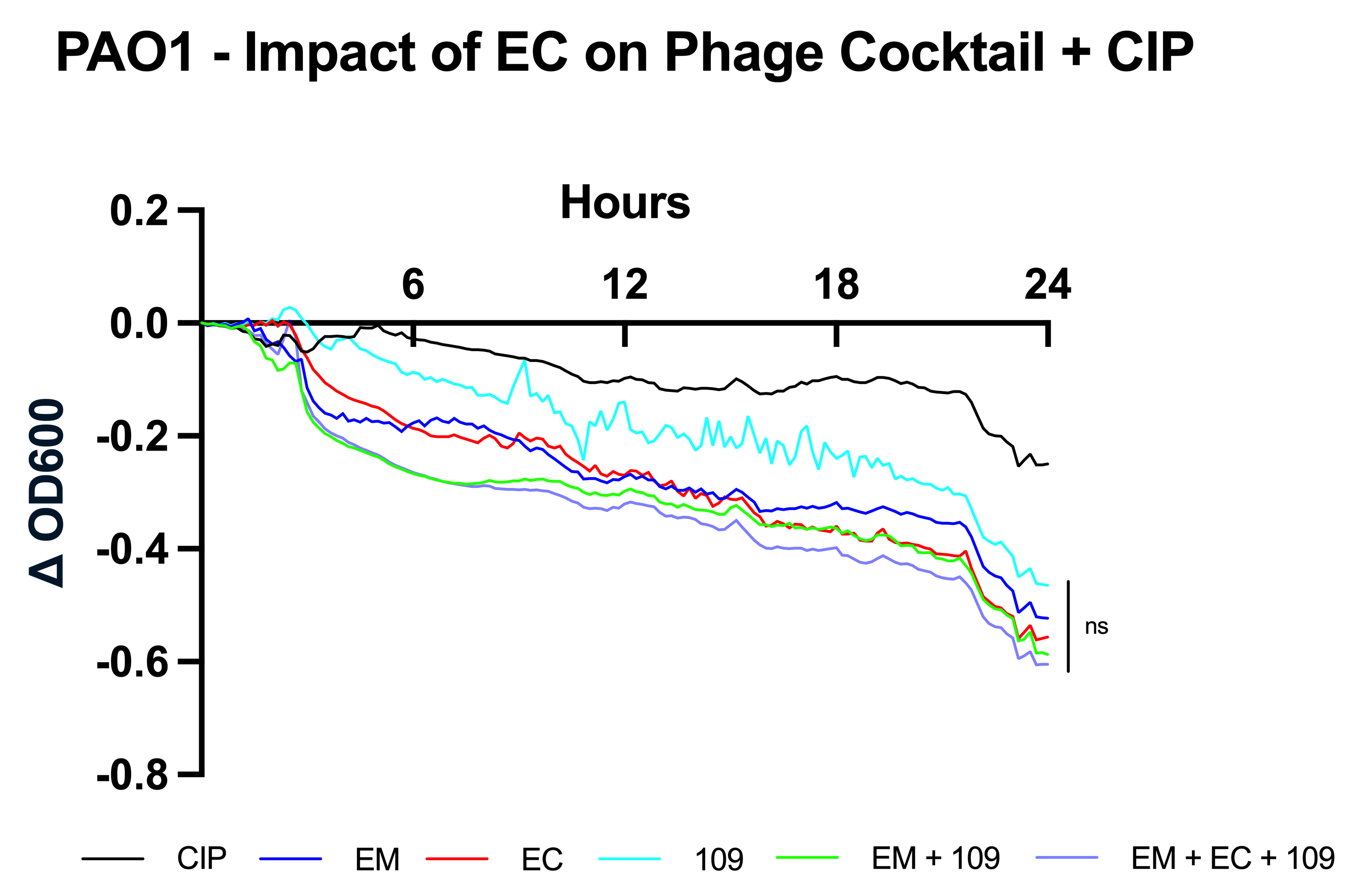** | |
| **Figure S1**: Twenty-four-hour continuous relative biofilm growth suppression following 24-hour biofilm maturation phase. Data are presented as the difference in OD600 values relative to the growth control after normalization to starting OD600 to account for differences in biofilm production between strains. Each plot illustrates growth of PAO1 or PA14 treated with respective phage ± CIP relative to growth control. Phages were dosed at MOI ≅ 1 and CIP was dosed at 0.5x MIC for each organism. Asterisks denote significant (p<0.05) differences between treatments, whilst “ns” signifies non-significance. All experiments performed in triplicate. **A.** Biofilm of *P. aeruginosa* strain PAO1 treated with various phage(s) without CIP **B.** P. aeruginosa strain PA14 treated with various phages without CIP **C.** PAO1 treated with various phage(s) in combination with CIP **D.** PA14 treated with various phage(s) in combination with CIP | |

| **A. PA14: EM + 109**  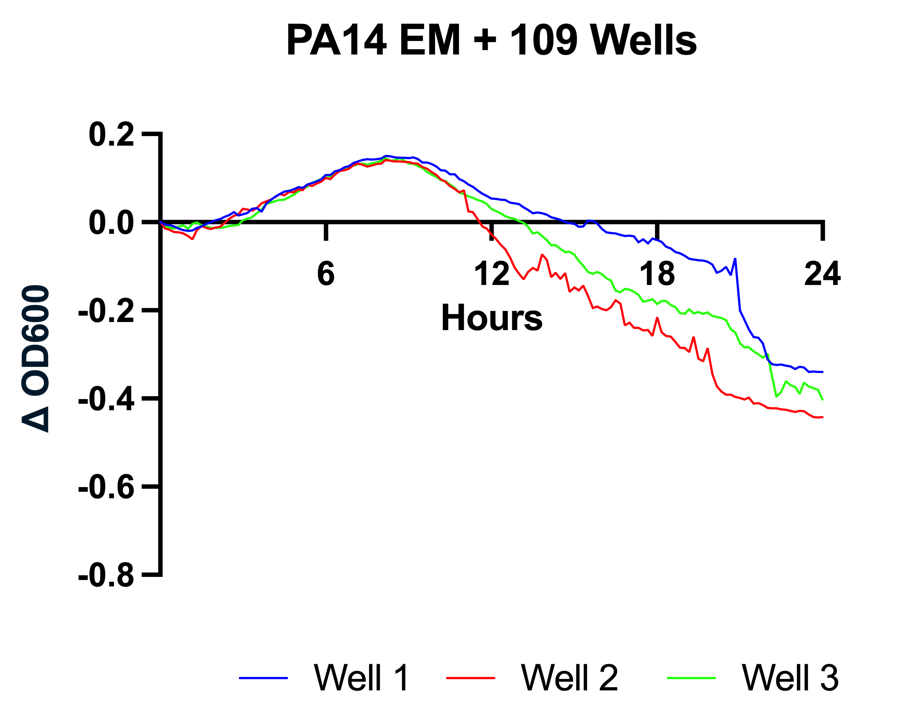 | 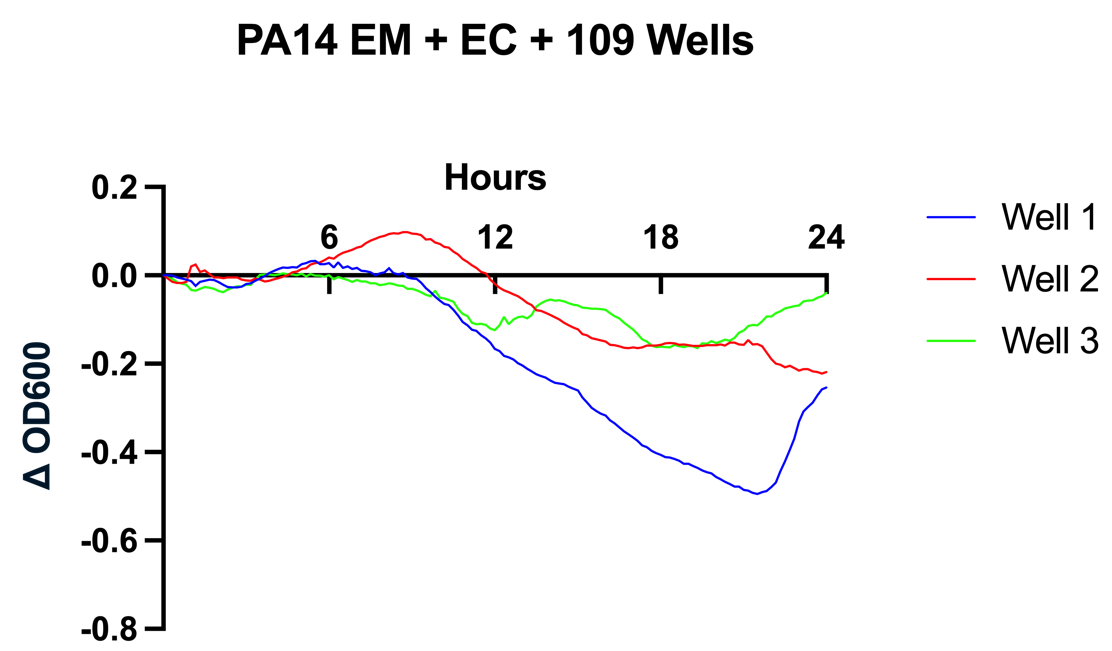  **B. PA14: EM + EC + 109** |
| --- | --- |
| 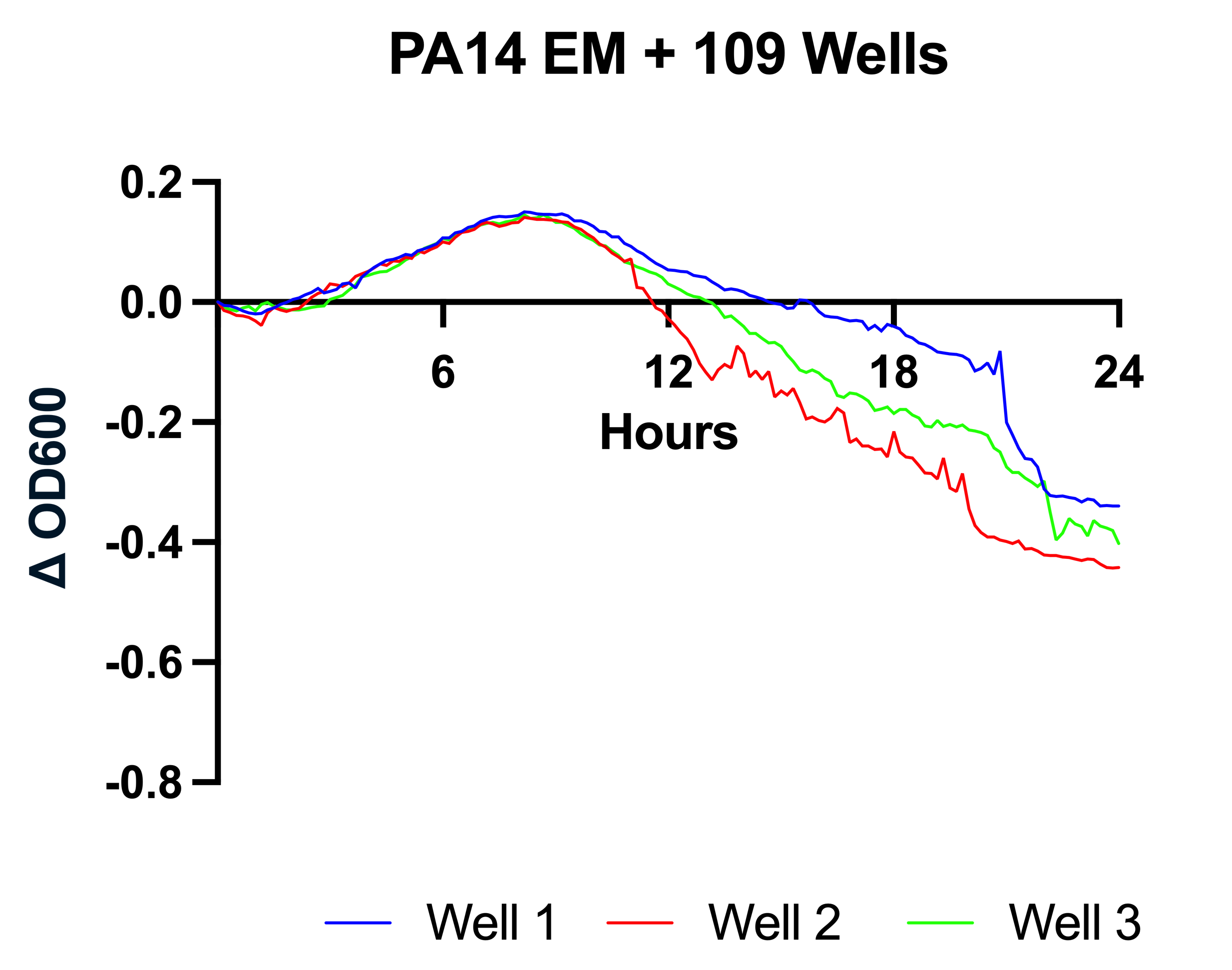 | |
| **Figure S2**: Twenty-four-hour continuous relative biofilm growth suppression following 24-hour biofilm maturation phase. Data are presented as the difference in OD600 values relative to the growth control after normalization to starting OD600 to account for differences in biofilm production between strains. Each plot illustrates growth of biofilm of *P. aeruginosa* strain PA14 treated with respective phage combination relative to growth control to illustrate the impact of Phage EC on the antibiofilm activity of the phage cocktail. Phages were dosed at MOI ≅ 1. Each line denotes an individual experiment assessing phage antibiofilm activity, with three identical experiments included in each plot. **A.** Biofilm of PA14 treated with a two phage cocktail of EM + 109, excluding EC **B.** Biofilm of PA14 treated with a three phage cocktail of EM + EC + 109. | |

| 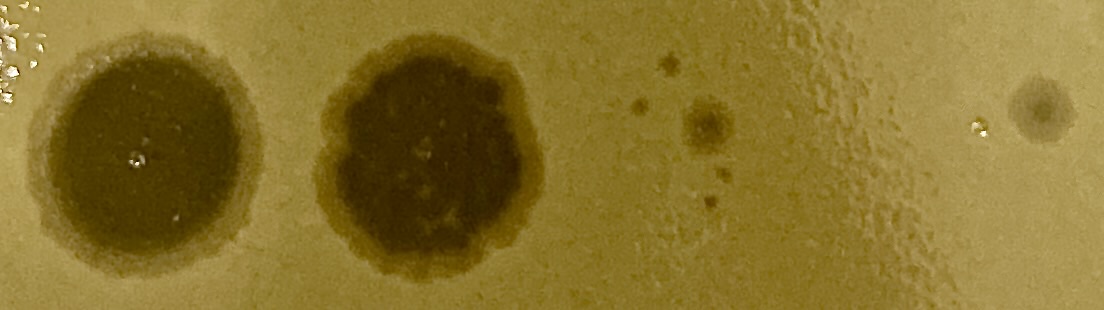 **+2**  **+4**  **+6**  **+8** |
| --- |
| **Figure S3:** Plaque morphology of bacteriophage EM-T3762627-2_AH (EM). Serial 10-fold dilutions of phage EM were assayed on lawns of its host strain, Pseudomonas aeruginosa EM-T3762627-2 (provided by Jose Alexander, AdventHealth Orlando, Winter Springs, FL, USA), via agar plaque assay. Plaques were characterized by clear centers surrounded by turbid zones. |

**Table S1:** Statistical evaluation of spectrophotometric analysis of phage-antibiotic activity against established biofilms

| Test details | Mean 1 | Mean 2 | Mean Diff. | Adjusted P Value |
| --- | --- | --- | --- | --- |
| PAO1 EM vs. PAO1 TRIPLE CIP | -0.426 | -0.604 | 0.178 | 0.7728 |
| PAO1 EM vs. PAO1 EC | -0.426 | -0.313 | -0.113 | 0.9997 |
| PAO1 EM vs. PAO1 109 | -0.426 | -0.367 | -0.060 | >0.9999 |
| PAO1 EM vs. PAO1 EM + 109 | -0.426 | -0.417 | -0.009 | >0.9999 |
| PAO1 EM vs. PAO1 CIP | -0.426 | -0.250 | -0.176 | 0.787 |
| PAO1 EM vs. PAO1 EM 109 CIP | -0.426 | -0.587 | 0.161 | 0.8961 |
| PAO1 EM vs. PAO1 EM CIP | -0.426 | -0.523 | 0.096 | >0.9999 |
| PAO1 EM vs. PAO1 EM EC CIP | -0.426 | -0.629 | 0.202 | 0.5354 |
| PAO1 EM vs. PAO1 EC CIP | -0.426 | -0.557 | 0.130 | 0.9897 |
| PAO1 EM vs. PAO1 EC 109 CIP | -0.426 | -0.583 | 0.157 | 0.9173 |
| PAO1 EM vs. PAO1 109 CIP | -0.426 | -0.465 | 0.038 | >0.9999 |
| PAO1 EM vs. PAO1 EC 109 | -0.426 | -0.490 | 0.064 | >0.9999 |
| PAO1 EM vs. PAO1 Triple | -0.426 | -0.524 | 0.098 | 0.9999 |
| PAO1 EM vs. PA14 EM | -0.426 | -0.377 | -0.050 | >0.9999 |
| PAO1 EM vs. PA14 Triple CIP | -0.426 | -0.657 | 0.231 | 0.2772 |
| PAO1 EM vs. PA14 EC | -0.426 | -0.038 | -0.388 | 0.0004 |
| PAO1 EM vs. PA14 109 | -0.426 | -0.045 | -0.381 | 0.0006 |
| PAO1 EM vs. PA14 EM 109 | -0.426 | -0.394 | -0.032 | >0.9999 |
| PAO1 EM vs. PA14 CIP | -0.426 | -0.459 | 0.033 | >0.9999 |
| PAO1 EM vs. PA14 EM 109 CIP | -0.426 | -0.721 | 0.295 | 0.0305 |
| PAO1 EM vs. PA14 EM CIP | -0.426 | -0.670 | 0.243 | 0.1922 |
| PAO1 EM vs. PA14 EM EC CIP | -0.426 | -0.672 | 0.246 | 0.1771 |
| PAO1 EM vs. PA14 EC CIP | -0.426 | -0.635 | 0.209 | 0.4718 |
| PAO1 EM vs. PA14 EC 109 CIP | -0.426 | -0.661 | 0.235 | 0.2481 |
| PAO1 EM vs. PA14 109 CIP | -0.426 | -0.623 | 0.196 | 0.5964 |
| PAO1 EM vs. PA14 109 EC | -0.426 | -0.100 | -0.327 | 0.0078 |
| PAO1 EM vs. PA14 TRIPLE | -0.426 | -0.171 | -0.256 | 0.1297 |
| PAO1 TRIPLE CIP vs. PAO1 EC | -0.604 | -0.313 | -0.291 | 0.0353 |
| PAO1 TRIPLE CIP vs. PAO1 109 | -0.604 | -0.367 | -0.238 | 0.0918 |
| PAO1 TRIPLE CIP vs. PAO1 EM + 109 | -0.604 | -0.417 | -0.187 | 0.4642 |
| PAO1 TRIPLE CIP vs. PAO1 CIP | -0.604 | -0.250 | -0.354 | 0.0003 |
| PAO1 TRIPLE CIP vs. PAO1 EM 109 CIP | -0.604 | -0.587 | -0.017 | >0.9999 |
| PAO1 TRIPLE CIP vs. PAO1 EM CIP | -0.604 | -0.523 | -0.082 | >0.9999 |
| PAO1 TRIPLE CIP vs. PAO1 EM EC CIP | -0.604 | -0.629 | 0.024 | >0.9999 |
| PAO1 TRIPLE CIP vs. PAO1 EC CIP | -0.604 | -0.557 | -0.048 | >0.9999 |
| PAO1 TRIPLE CIP vs. PAO1 EC 109 CIP | -0.604 | -0.583 | -0.021 | >0.9999 |
| PAO1 TRIPLE CIP vs. PAO1 109 CIP | -0.604 | -0.465 | -0.140 | 0.9214 |
| PAO1 TRIPLE CIP vs. PAO1 EC 109 | -0.604 | -0.490 | -0.114 | 0.9921 |
| PAO1 TRIPLE CIP vs. PAO1 Triple | -0.604 | -0.524 | -0.080 | >0.9999 |
| PAO1 TRIPLE CIP vs. PA14 EM | -0.604 | -0.377 | -0.228 | 0.1347 |
| PAO1 TRIPLE CIP vs. PA14 Triple CIP | -0.604 | -0.657 | 0.053 | >0.9999 |
| PAO1 TRIPLE CIP vs. PA14 EC | -0.604 | -0.038 | -0.566 | <0.0001 |
| PAO1 TRIPLE CIP vs. PA14 109 | -0.604 | -0.045 | -0.559 | <0.0001 |
| PAO1 TRIPLE CIP vs. PA14 EM 109 | -0.604 | -0.394 | -0.210 | 0.2473 |
| PAO1 TRIPLE CIP vs. PA14 CIP | -0.604 | -0.459 | -0.145 | 0.8875 |
| PAO1 TRIPLE CIP vs. PA14 EM 109 CIP | -0.604 | -0.721 | 0.117 | 0.9896 |
| PAO1 TRIPLE CIP vs. PA14 EM CIP | -0.604 | -0.670 | 0.065 | >0.9999 |
| PAO1 TRIPLE CIP vs. PA14 EM EC CIP | -0.604 | -0.672 | 0.068 | >0.9999 |
| PAO1 TRIPLE CIP vs. PA14 EC CIP | -0.604 | -0.635 | 0.031 | >0.9999 |
| PAO1 TRIPLE CIP vs. PA14 EC 109 CIP | -0.604 | -0.661 | 0.057 | >0.9999 |
| PAO1 TRIPLE CIP vs. PA14 109 CIP | -0.604 | -0.623 | 0.018 | >0.9999 |
| PAO1 TRIPLE CIP vs. PA14 109 EC | -0.604 | -0.100 | -0.505 | <0.0001 |
| PAO1 TRIPLE CIP vs. PA14 TRIPLE | -0.604 | -0.171 | -0.434 | <0.0001 |
| PAO1 EC vs. PAO1 109 | -0.313 | -0.367 | 0.053 | >0.9999 |
| PAO1 EC vs. PAO1 EM + 109 | -0.313 | -0.417 | 0.104 | 0.9997 |
| PAO1 EC vs. PAO1 CIP | -0.313 | -0.250 | -0.063 | >0.9999 |
| PAO1 EC vs. PAO1 EM 109 CIP | -0.313 | -0.587 | 0.274 | 0.068 |
| PAO1 EC vs. PAO1 EM CIP | -0.313 | -0.523 | 0.209 | 0.4653 |
| PAO1 EC vs. PAO1 EM EC CIP | -0.313 | -0.629 | 0.315 | 0.0128 |
| PAO1 EC vs. PAO1 EC CIP | -0.313 | -0.557 | 0.243 | 0.1923 |
| PAO1 EC vs. PAO1 EC 109 CIP | -0.313 | -0.583 | 0.270 | 0.0787 |
| PAO1 EC vs. PAO1 109 CIP | -0.313 | -0.465 | 0.151 | 0.9421 |
| PAO1 EC vs. PAO1 EC 109 | -0.313 | -0.490 | 0.177 | 0.7842 |
| PAO1 EC vs. PAO1 Triple | -0.313 | -0.524 | 0.211 | 0.4522 |
| PAO1 EC vs. PA14 EM | -0.313 | -0.377 | 0.063 | >0.9999 |
| PAO1 EC vs. PA14 Triple CIP | -0.313 | -0.657 | 0.344 | 0.0036 |
| PAO1 EC vs. PA14 EC | -0.313 | -0.038 | -0.275 | 0.0655 |
| PAO1 EC vs. PA14 109 | -0.313 | -0.045 | -0.268 | 0.0846 |
| PAO1 EC vs. PA14 EM 109 | -0.313 | -0.394 | 0.081 | >0.9999 |
| PAO1 EC vs. PA14 CIP | -0.313 | -0.459 | 0.146 | 0.9611 |
| PAO1 EC vs. PA14 EM 109 CIP | -0.313 | -0.721 | 0.408 | 0.0002 |
| PAO1 EC vs. PA14 EM CIP | -0.313 | -0.670 | 0.356 | 0.002 |
| PAO1 EC vs. PA14 EM EC CIP | -0.313 | -0.672 | 0.359 | 0.0017 |
| PAO1 EC vs. PA14 EC CIP | -0.313 | -0.635 | 0.322 | 0.0097 |
| PAO1 EC vs. PA14 EC 109 CIP | -0.313 | -0.661 | 0.348 | 0.003 |
| PAO1 EC vs. PA14 109 CIP | -0.313 | -0.623 | 0.309 | 0.0166 |
| PAO1 EC vs. PA14 109 EC | -0.313 | -0.100 | -0.214 | 0.4234 |
| PAO1 EC vs. PA14 TRIPLE | -0.313 | -0.171 | -0.143 | 0.9692 |
| PAO1 109 vs. PAO1 EM + 109 | -0.367 | -0.417 | 0.050 | >0.9999 |
| PAO1 109 vs. PAO1 CIP | -0.367 | -0.250 | -0.117 | 0.9896 |
| PAO1 109 vs. PAO1 EM 109 CIP | -0.367 | -0.587 | 0.221 | 0.1732 |
| PAO1 109 vs. PAO1 EM CIP | -0.367 | -0.523 | 0.156 | 0.8029 |
| PAO1 109 vs. PAO1 EM EC CIP | -0.367 | -0.629 | 0.262 | 0.0327 |
| PAO1 109 vs. PAO1 EC CIP | -0.367 | -0.557 | 0.190 | 0.4352 |
| PAO1 109 vs. PAO1 EC 109 CIP | -0.367 | -0.583 | 0.217 | 0.1987 |
| PAO1 109 vs. PAO1 109 CIP | -0.367 | -0.465 | 0.098 | 0.9992 |
| PAO1 109 vs. PAO1 EC 109 | -0.367 | -0.490 | 0.123 | 0.9792 |
| PAO1 109 vs. PAO1 Triple | -0.367 | -0.524 | 0.157 | 0.7906 |
| PAO1 109 vs. PA14 EM | -0.367 | -0.377 | 0.010 | >0.9999 |
| PAO1 109 vs. PA14 Triple CIP | -0.367 | -0.657 | 0.290 | 0.0085 |
| PAO1 109 vs. PA14 EC | -0.367 | -0.038 | -0.328 | 0.0012 |
| PAO1 109 vs. PA14 109 | -0.367 | -0.045 | -0.321 | 0.0017 |
| PAO1 109 vs. PA14 EM 109 | -0.367 | -0.394 | 0.028 | >0.9999 |
| PAO1 109 vs. PA14 CIP | -0.367 | -0.459 | 0.092 | 0.9997 |
| PAO1 109 vs. PA14 EM 109 CIP | -0.367 | -0.721 | 0.354 | 0.0003 |
| PAO1 109 vs. PA14 EM CIP | -0.367 | -0.670 | 0.303 | 0.0045 |
| PAO1 109 vs. PA14 EM EC CIP | -0.367 | -0.672 | 0.306 | 0.0039 |
| PAO1 109 vs. PA14 EC CIP | -0.367 | -0.635 | 0.268 | 0.0245 |
| PAO1 109 vs. PA14 EC 109 CIP | -0.367 | -0.661 | 0.294 | 0.007 |
| PAO1 109 vs. PA14 109 CIP | -0.367 | -0.623 | 0.256 | 0.0427 |
| PAO1 109 vs. PA14 109 EC | -0.367 | -0.100 | -0.267 | 0.0261 |
| PAO1 109 vs. PA14 TRIPLE | -0.367 | -0.171 | -0.196 | 0.3729 |
| PAO1 EM + 109 vs. PAO1 CIP | -0.417 | -0.250 | -0.167 | 0.6925 |
| PAO1 EM + 109 vs. PAO1 EM 109 CIP | -0.417 | -0.587 | 0.170 | 0.6559 |
| PAO1 EM + 109 vs. PAO1 EM CIP | -0.417 | -0.523 | 0.106 | 0.9974 |
| PAO1 EM + 109 vs. PAO1 EM EC CIP | -0.417 | -0.629 | 0.212 | 0.2344 |
| PAO1 EM + 109 vs. PAO1 EC CIP | -0.417 | -0.557 | 0.140 | 0.9214 |
| PAO1 EM + 109 vs. PAO1 EC 109 CIP | -0.417 | -0.583 | 0.166 | 0.6997 |
| PAO1 EM + 109 vs. PAO1 109 CIP | -0.417 | -0.465 | 0.048 | >0.9999 |
| PAO1 EM + 109 vs. PAO1 EC 109 | -0.417 | -0.490 | 0.073 | >0.9999 |
| PAO1 EM + 109 vs. PAO1 Triple | -0.417 | -0.524 | 0.107 | 0.9969 |
| PAO1 EM + 109 vs. PA14 EM | -0.417 | -0.377 | -0.040 | >0.9999 |
| PAO1 EM + 109 vs. PA14 Triple CIP | -0.417 | -0.657 | 0.240 | 0.0836 |
| PAO1 EM + 109 vs. PA14 EC | -0.417 | -0.038 | -0.379 | <0.0001 |
| PAO1 EM + 109 vs. PA14 109 | -0.417 | -0.045 | -0.372 | 0.0001 |
| PAO1 EM + 109 vs. PA14 EM 109 | -0.417 | -0.394 | -0.023 | >0.9999 |
| PAO1 EM + 109 vs. PA14 CIP | -0.417 | -0.459 | 0.042 | >0.9999 |
| PAO1 EM + 109 vs. PA14 EM 109 CIP | -0.417 | -0.721 | 0.304 | 0.0043 |
| PAO1 EM + 109 vs. PA14 EM CIP | -0.417 | -0.670 | 0.253 | 0.0493 |
| PAO1 EM + 109 vs. PA14 EM EC CIP | -0.417 | -0.672 | 0.255 | 0.0439 |
| PAO1 EM + 109 vs. PA14 EC CIP | -0.417 | -0.635 | 0.218 | 0.1899 |
| PAO1 EM + 109 vs. PA14 EC 109 CIP | -0.417 | -0.661 | 0.244 | 0.0711 |
| PAO1 EM + 109 vs. PA14 109 CIP | -0.417 | -0.623 | 0.206 | 0.2827 |
| PAO1 EM + 109 vs. PA14 109 EC | -0.417 | -0.100 | -0.317 | 0.0021 |
| PAO1 EM + 109 vs. PA14 TRIPLE | -0.417 | -0.171 | -0.246 | 0.0645 |
| PAO1 CIP vs. PAO1 EM 109 CIP | -0.250 | -0.587 | 0.337 | 0.0007 |
| PAO1 CIP vs. PAO1 EM CIP | -0.250 | -0.523 | 0.273 | 0.0201 |
| PAO1 CIP vs. PAO1 EM EC CIP | -0.250 | -0.629 | 0.379 | <0.0001 |
| PAO1 CIP vs. PAO1 EC CIP | -0.250 | -0.557 | 0.307 | 0.0037 |
| PAO1 CIP vs. PAO1 EC 109 CIP | -0.250 | -0.583 | 0.333 | 0.0009 |
| PAO1 CIP vs. PAO1 109 CIP | -0.250 | -0.465 | 0.215 | 0.2125 |
| PAO1 CIP vs. PAO1 EC 109 | -0.250 | -0.490 | 0.240 | 0.0836 |
| PAO1 CIP vs. PAO1 Triple | -0.250 | -0.524 | 0.274 | 0.0188 |
| PAO1 CIP vs. PA14 EM | -0.250 | -0.377 | 0.127 | 0.9716 |
| PAO1 CIP vs. PA14 Triple CIP | -0.250 | -0.657 | 0.407 | <0.0001 |
| PAO1 CIP vs. PA14 EC | -0.250 | -0.038 | -0.212 | 0.2345 |
| PAO1 CIP vs. PA14 109 | -0.250 | -0.045 | -0.205 | 0.2915 |
| PAO1 CIP vs. PA14 EM 109 | -0.250 | -0.394 | 0.144 | 0.8939 |
| PAO1 CIP vs. PA14 CIP | -0.250 | -0.459 | 0.209 | 0.2551 |
| PAO1 CIP vs. PA14 EM 109 CIP | -0.250 | -0.721 | 0.471 | <0.0001 |
| PAO1 CIP vs. PA14 EM CIP | -0.250 | -0.670 | 0.420 | <0.0001 |
| PAO1 CIP vs. PA14 EM EC CIP | -0.250 | -0.672 | 0.422 | <0.0001 |
| PAO1 CIP vs. PA14 EC CIP | -0.250 | -0.635 | 0.385 | <0.0001 |
| PAO1 CIP vs. PA14 EC 109 CIP | -0.250 | -0.661 | 0.411 | <0.0001 |
| PAO1 CIP vs. PA14 109 CIP | -0.250 | -0.623 | 0.373 | <0.0001 |
| PAO1 CIP vs. PA14 109 EC | -0.250 | -0.100 | -0.150 | 0.8511 |
| PAO1 CIP vs. PA14 TRIPLE | -0.250 | -0.171 | -0.079 | >0.9999 |
| PAO1 EM 109 CIP vs. PAO1 EM CIP | -0.587 | -0.523 | -0.065 | >0.9999 |
| PAO1 EM 109 CIP vs. PAO1 EM EC CIP | -0.587 | -0.629 | 0.041 | >0.9999 |
| PAO1 EM 109 CIP vs. PAO1 EC CIP | -0.587 | -0.557 | -0.031 | >0.9999 |
| PAO1 EM 109 CIP vs. PAO1 EC 109 CIP | -0.587 | -0.583 | -0.004 | >0.9999 |
| PAO1 EM 109 CIP vs. PAO1 109 CIP | -0.587 | -0.465 | -0.123 | 0.9805 |
| PAO1 EM 109 CIP vs. PAO1 EC 109 | -0.587 | -0.490 | -0.097 | 0.9993 |
| PAO1 EM 109 CIP vs. PAO1 Triple | -0.587 | -0.524 | -0.063 | >0.9999 |
| PAO1 EM 109 CIP vs. PA14 EM | -0.587 | -0.377 | -0.211 | 0.2421 |
| PAO1 EM 109 CIP vs. PA14 Triple CIP | -0.587 | -0.657 | 0.070 | >0.9999 |
| PAO1 EM 109 CIP vs. PA14 EC | -0.587 | -0.038 | -0.549 | <0.0001 |
| PAO1 EM 109 CIP vs. PA14 109 | -0.587 | -0.045 | -0.542 | <0.0001 |
| PAO1 EM 109 CIP vs. PA14 EM 109 | -0.587 | -0.394 | -0.193 | 0.4036 |
| PAO1 EM 109 CIP vs. PA14 CIP | -0.587 | -0.459 | -0.128 | 0.9671 |
| PAO1 EM 109 CIP vs. PA14 EM 109 CIP | -0.587 | -0.721 | 0.134 | 0.949 |
| PAO1 EM 109 CIP vs. PA14 EM CIP | -0.587 | -0.670 | 0.082 | >0.9999 |
| PAO1 EM 109 CIP vs. PA14 EM EC CIP | -0.587 | -0.672 | 0.085 | >0.9999 |
| PAO1 EM 109 CIP vs. PA14 EC CIP | -0.587 | -0.635 | 0.048 | >0.9999 |
| PAO1 EM 109 CIP vs. PA14 EC 109 CIP | -0.587 | -0.661 | 0.074 | >0.9999 |
| PAO1 EM 109 CIP vs. PA14 109 CIP | -0.587 | -0.623 | 0.035 | >0.9999 |
| PAO1 EM 109 CIP vs. PA14 109 EC | -0.587 | -0.100 | -0.488 | <0.0001 |
| PAO1 EM 109 CIP vs. PA14 TRIPLE | -0.587 | -0.171 | -0.417 | <0.0001 |
| PAO1 EM CIP vs. PAO1 EM EC CIP | -0.523 | -0.629 | 0.106 | 0.9973 |
| PAO1 EM CIP vs. PAO1 EC CIP | -0.523 | -0.557 | 0.034 | >0.9999 |
| PAO1 EM CIP vs. PAO1 EC 109 CIP | -0.523 | -0.583 | 0.061 | >0.9999 |
| PAO1 EM CIP vs. PAO1 109 CIP | -0.523 | -0.465 | -0.058 | >0.9999 |
| PAO1 EM CIP vs. PAO1 EC 109 | -0.523 | -0.490 | -0.033 | >0.9999 |
| PAO1 EM CIP vs. PAO1 Triple | -0.523 | -0.524 | 0.001 | >0.9999 |
| PAO1 EM CIP vs. PA14 EM | -0.523 | -0.377 | -0.146 | 0.883 |
| PAO1 EM CIP vs. PA14 Triple CIP | -0.523 | -0.657 | 0.134 | 0.9463 |
| PAO1 EM CIP vs. PA14 EC | -0.523 | -0.038 | -0.484 | <0.0001 |
| PAO1 EM CIP vs. PA14 109 | -0.523 | -0.045 | -0.477 | <0.0001 |
| PAO1 EM CIP vs. PA14 EM 109 | -0.523 | -0.394 | -0.128 | 0.9671 |
| PAO1 EM CIP vs. PA14 CIP | -0.523 | -0.459 | -0.064 | >0.9999 |
| PAO1 EM CIP vs. PA14 EM 109 CIP | -0.523 | -0.721 | 0.198 | 0.3497 |
| PAO1 EM CIP vs. PA14 EM CIP | -0.523 | -0.670 | 0.147 | 0.8759 |
| PAO1 EM CIP vs. PA14 EM EC CIP | -0.523 | -0.672 | 0.150 | 0.8562 |
| PAO1 EM CIP vs. PA14 EC CIP | -0.523 | -0.635 | 0.112 | 0.9938 |
| PAO1 EM CIP vs. PA14 EC 109 CIP | -0.523 | -0.661 | 0.138 | 0.9282 |
| PAO1 EM CIP vs. PA14 109 CIP | -0.523 | -0.623 | 0.100 | 0.9989 |
| PAO1 EM CIP vs. PA14 109 EC | -0.523 | -0.100 | -0.423 | <0.0001 |
| PAO1 EM CIP vs. PA14 TRIPLE | -0.523 | -0.171 | -0.352 | 0.0003 |
| PAO1 EM EC CIP vs. PAO1 EC CIP | -0.629 | -0.557 | -0.072 | >0.9999 |
| PAO1 EM EC CIP vs. PAO1 EC 109 CIP | -0.629 | -0.583 | -0.045 | >0.9999 |
| PAO1 EM EC CIP vs. PAO1 109 CIP | -0.629 | -0.465 | -0.164 | 0.7244 |
| PAO1 EM EC CIP vs. PAO1 EC 109 | -0.629 | -0.490 | -0.139 | 0.9266 |
| PAO1 EM EC CIP vs. PAO1 Triple | -0.629 | -0.524 | -0.105 | 0.9978 |
| PAO1 EM EC CIP vs. PA14 EM | -0.629 | -0.377 | -0.252 | 0.0508 |
| PAO1 EM EC CIP vs. PA14 Triple CIP | -0.629 | -0.657 | 0.028 | >0.9999 |
| PAO1 EM EC CIP vs. PA14 EC | -0.629 | -0.038 | -0.590 | <0.0001 |
| PAO1 EM EC CIP vs. PA14 109 | -0.629 | -0.045 | -0.583 | <0.0001 |
| PAO1 EM EC CIP vs. PA14 EM 109 | -0.629 | -0.394 | -0.234 | 0.1046 |
| PAO1 EM EC CIP vs. PA14 CIP | -0.629 | -0.459 | -0.170 | 0.6634 |
| PAO1 EM EC CIP vs. PA14 EM 109 CIP | -0.629 | -0.721 | 0.092 | 0.9997 |
| PAO1 EM EC CIP vs. PA14 EM CIP | -0.629 | -0.670 | 0.041 | >0.9999 |
| PAO1 EM EC CIP vs. PA14 EM EC CIP | -0.629 | -0.672 | 0.044 | >0.9999 |
| PAO1 EM EC CIP vs. PA14 EC CIP | -0.629 | -0.635 | 0.006 | >0.9999 |
| PAO1 EM EC CIP vs. PA14 EC 109 CIP | -0.629 | -0.661 | 0.032 | >0.9999 |
| PAO1 EM EC CIP vs. PA14 109 CIP | -0.629 | -0.623 | -0.006 | >0.9999 |
| PAO1 EM EC CIP vs. PA14 109 EC | -0.629 | -0.100 | -0.529 | <0.0001 |
| PAO1 EM EC CIP vs. PA14 TRIPLE | -0.629 | -0.171 | -0.458 | <0.0001 |
| PAO1 EC CIP vs. PAO1 EC 109 CIP | -0.557 | -0.583 | 0.027 | >0.9999 |
| PAO1 EC CIP vs. PAO1 109 CIP | -0.557 | -0.465 | -0.092 | 0.9997 |
| PAO1 EC CIP vs. PAO1 EC 109 | -0.557 | -0.490 | -0.067 | >0.9999 |
| PAO1 EC CIP vs. PAO1 Triple | -0.557 | -0.524 | -0.033 | >0.9999 |
| PAO1 EC CIP vs. PA14 EM | -0.557 | -0.377 | -0.180 | 0.5465 |
| PAO1 EC CIP vs. PA14 Triple CIP | -0.557 | -0.657 | 0.100 | 0.9988 |
| PAO1 EC CIP vs. PA14 EC | -0.557 | -0.038 | -0.518 | <0.0001 |
| PAO1 EC CIP vs. PA14 109 | -0.557 | -0.045 | -0.511 | <0.0001 |
| PAO1 EC CIP vs. PA14 EM 109 | -0.557 | -0.394 | -0.162 | 0.7417 |
| PAO1 EC CIP vs. PA14 CIP | -0.557 | -0.459 | -0.098 | 0.9993 |
| PAO1 EC CIP vs. PA14 EM 109 CIP | -0.557 | -0.721 | 0.164 | 0.7209 |
| PAO1 EC CIP vs. PA14 EM CIP | -0.557 | -0.670 | 0.113 | 0.9932 |
| PAO1 EC CIP vs. PA14 EM EC CIP | -0.557 | -0.672 | 0.116 | 0.9907 |
| PAO1 EC CIP vs. PA14 EC CIP | -0.557 | -0.635 | 0.078 | >0.9999 |
| PAO1 EC CIP vs. PA14 EC 109 CIP | -0.557 | -0.661 | 0.104 | 0.9979 |
| PAO1 EC CIP vs. PA14 109 CIP | -0.557 | -0.623 | 0.066 | >0.9999 |
| PAO1 EC CIP vs. PA14 109 EC | -0.557 | -0.100 | -0.457 | <0.0001 |
| PAO1 EC CIP vs. PA14 TRIPLE | -0.557 | -0.171 | -0.386 | <0.0001 |
| PAO1 EC 109 CIP vs. PAO1 109 CIP | -0.583 | -0.465 | -0.119 | 0.9871 |
| PAO1 EC 109 CIP vs. PAO1 EC 109 | -0.583 | -0.490 | -0.093 | 0.9997 |
| PAO1 EC 109 CIP vs. PAO1 Triple | -0.583 | -0.524 | -0.059 | >0.9999 |
| PAO1 EC 109 CIP vs. PA14 EM | -0.583 | -0.377 | -0.207 | 0.2744 |
| PAO1 EC 109 CIP vs. PA14 Triple CIP | -0.583 | -0.657 | 0.074 | >0.9999 |
| PAO1 EC 109 CIP vs. PA14 EC | -0.583 | -0.038 | -0.545 | <0.0001 |
| PAO1 EC 109 CIP vs. PA14 109 | -0.583 | -0.045 | -0.538 | <0.0001 |
| PAO1 EC 109 CIP vs. PA14 EM 109 | -0.583 | -0.394 | -0.189 | 0.4461 |
| PAO1 EC 109 CIP vs. PA14 CIP | -0.583 | -0.459 | -0.124 | 0.9771 |
| PAO1 EC 109 CIP vs. PA14 EM 109 CIP | -0.583 | -0.721 | 0.138 | 0.9315 |
| PAO1 EC 109 CIP vs. PA14 EM CIP | -0.583 | -0.670 | 0.086 | >0.9999 |
| PAO1 EC 109 CIP vs. PA14 EM EC CIP | -0.583 | -0.672 | 0.089 | 0.9998 |
| PAO1 EC 109 CIP vs. PA14 EC CIP | -0.583 | -0.635 | 0.052 | >0.9999 |
| PAO1 EC 109 CIP vs. PA14 EC 109 CIP | -0.583 | -0.661 | 0.078 | >0.9999 |
| PAO1 EC 109 CIP vs. PA14 109 CIP | -0.583 | -0.623 | 0.039 | >0.9999 |
| PAO1 EC 109 CIP vs. PA14 109 EC | -0.583 | -0.100 | -0.484 | <0.0001 |
| PAO1 EC 109 CIP vs. PA14 TRIPLE | -0.583 | -0.171 | -0.413 | <0.0001 |
| PAO1 109 CIP vs. PAO1 EC 109 | -0.465 | -0.490 | 0.025 | >0.9999 |
| PAO1 109 CIP vs. PAO1 Triple | -0.465 | -0.524 | 0.059 | >0.9999 |
| PAO1 109 CIP vs. PA14 EM | -0.465 | -0.377 | -0.088 | 0.9999 |
| PAO1 109 CIP vs. PA14 Triple CIP | -0.465 | -0.657 | 0.192 | 0.4104 |
| PAO1 109 CIP vs. PA14 EC | -0.465 | -0.038 | -0.426 | <0.0001 |
| PAO1 109 CIP vs. PA14 109 | -0.465 | -0.045 | -0.419 | <0.0001 |
| PAO1 109 CIP vs. PA14 EM 109 | -0.465 | -0.394 | -0.070 | >0.9999 |
| PAO1 109 CIP vs. PA14 CIP | -0.465 | -0.459 | -0.006 | >0.9999 |
| PAO1 109 CIP vs. PA14 EM 109 CIP | -0.465 | -0.721 | 0.256 | 0.0421 |
| PAO1 109 CIP vs. PA14 EM CIP | -0.465 | -0.670 | 0.205 | 0.2885 |
| PAO1 109 CIP vs. PA14 EM EC CIP | -0.465 | -0.672 | 0.208 | 0.2659 |
| PAO1 109 CIP vs. PA14 EC CIP | -0.465 | -0.635 | 0.170 | 0.6558 |
| PAO1 109 CIP vs. PA14 EC 109 CIP | -0.465 | -0.661 | 0.196 | 0.3694 |
| PAO1 109 CIP vs. PA14 109 CIP | -0.465 | -0.623 | 0.158 | 0.7843 |
| PAO1 109 CIP vs. PA14 109 EC | -0.465 | -0.100 | -0.365 | 0.0002 |
| PAO1 109 CIP vs. PA14 TRIPLE | -0.465 | -0.171 | -0.294 | 0.0071 |
| PAO1 EC 109 vs. PAO1 Triple | -0.490 | -0.524 | 0.034 | >0.9999 |
| PAO1 EC 109 vs. PA14 EM | -0.490 | -0.377 | -0.113 | 0.993 |
| PAO1 EC 109 vs. PA14 Triple CIP | -0.490 | -0.657 | 0.167 | 0.6924 |
| PAO1 EC 109 vs. PA14 EC | -0.490 | -0.038 | -0.452 | <0.0001 |
| PAO1 EC 109 vs. PA14 109 | -0.490 | -0.045 | -0.445 | <0.0001 |
| PAO1 EC 109 vs. PA14 EM 109 | -0.490 | -0.394 | -0.096 | 0.9995 |
| PAO1 EC 109 vs. PA14 CIP | -0.490 | -0.459 | -0.031 | >0.9999 |
| PAO1 EC 109 vs. PA14 EM 109 CIP | -0.490 | -0.721 | 0.231 | 0.1188 |
| PAO1 EC 109 vs. PA14 EM CIP | -0.490 | -0.670 | 0.180 | 0.5501 |
| PAO1 EC 109 vs. PA14 EM EC CIP | -0.490 | -0.672 | 0.182 | 0.5199 |
| PAO1 EC 109 vs. PA14 EC CIP | -0.490 | -0.635 | 0.145 | 0.8896 |
| PAO1 EC 109 vs. PA14 EC 109 CIP | -0.490 | -0.661 | 0.171 | 0.6484 |
| PAO1 EC 109 vs. PA14 109 CIP | -0.490 | -0.623 | 0.133 | 0.9529 |
| PAO1 EC 109 vs. PA14 109 EC | -0.490 | -0.100 | -0.390 | <0.0001 |
| PAO1 EC 109 vs. PA14 TRIPLE | -0.490 | -0.171 | -0.319 | 0.0019 |
| PAO1 Triple vs. PA14 EM | -0.524 | -0.377 | -0.147 | 0.8736 |
| PAO1 Triple vs. PA14 Triple CIP | -0.524 | -0.657 | 0.133 | 0.9516 |
| PAO1 Triple vs. PA14 EC | -0.524 | -0.038 | -0.486 | <0.0001 |
| PAO1 Triple vs. PA14 109 | -0.524 | -0.045 | -0.479 | <0.0001 |
| PAO1 Triple vs. PA14 EM 109 | -0.524 | -0.394 | -0.130 | 0.9631 |
| PAO1 Triple vs. PA14 CIP | -0.524 | -0.459 | -0.065 | >0.9999 |
| PAO1 Triple vs. PA14 EM 109 CIP | -0.524 | -0.721 | 0.197 | 0.3628 |
| PAO1 Triple vs. PA14 EM CIP | -0.524 | -0.670 | 0.146 | 0.8851 |
| PAO1 Triple vs. PA14 EM EC CIP | -0.524 | -0.672 | 0.148 | 0.8663 |
| PAO1 Triple vs. PA14 EC CIP | -0.524 | -0.635 | 0.111 | 0.9947 |
| PAO1 Triple vs. PA14 EC 109 CIP | -0.524 | -0.661 | 0.137 | 0.9347 |
| PAO1 Triple vs. PA14 109 CIP | -0.524 | -0.623 | 0.099 | 0.9991 |
| PAO1 Triple vs. PA14 109 EC | -0.524 | -0.100 | -0.424 | <0.0001 |
| PAO1 Triple vs. PA14 TRIPLE | -0.524 | -0.171 | -0.353 | 0.0003 |
| PA14 EM vs. PA14 Triple CIP | -0.377 | -0.657 | 0.280 | 0.0139 |
| PA14 EM vs. PA14 EC | -0.377 | -0.038 | -0.338 | 0.0007 |
| PA14 EM vs. PA14 109 | -0.377 | -0.045 | -0.331 | 0.001 |
| PA14 EM vs. PA14 EM 109 | -0.377 | -0.394 | 0.018 | >0.9999 |
| PA14 EM vs. PA14 CIP | -0.377 | -0.459 | 0.082 | >0.9999 |
| PA14 EM vs. PA14 EM 109 CIP | -0.377 | -0.721 | 0.344 | 0.0005 |
| PA14 EM vs. PA14 EM CIP | -0.377 | -0.670 | 0.293 | 0.0075 |
| PA14 EM vs. PA14 EM EC CIP | -0.377 | -0.672 | 0.296 | 0.0065 |
| PA14 EM vs. PA14 EC CIP | -0.377 | -0.635 | 0.258 | 0.0385 |
| PA14 EM vs. PA14 EC 109 CIP | -0.377 | -0.661 | 0.284 | 0.0115 |
| PA14 EM vs. PA14 109 CIP | -0.377 | -0.623 | 0.246 | 0.0654 |
| PA14 EM vs. PA14 109 EC | -0.377 | -0.100 | -0.277 | 0.0163 |
| PA14 EM vs. PA14 TRIPLE | -0.377 | -0.171 | -0.206 | 0.2799 |
| PA14 Triple CIP vs. PA14 EC | -0.657 | -0.038 | -0.619 | <0.0001 |
| PA14 Triple CIP vs. PA14 109 | -0.657 | -0.045 | -0.612 | <0.0001 |
| PA14 Triple CIP vs. PA14 EM 109 | -0.657 | -0.394 | -0.263 | 0.0318 |
| PA14 Triple CIP vs. PA14 CIP | -0.657 | -0.459 | -0.198 | 0.3531 |
| PA14 Triple CIP vs. PA14 EM 109 CIP | -0.657 | -0.721 | 0.064 | >0.9999 |
| PA14 Triple CIP vs. PA14 EM CIP | -0.657 | -0.670 | 0.013 | >0.9999 |
| PA14 Triple CIP vs. PA14 EM EC CIP | -0.657 | -0.672 | 0.015 | >0.9999 |
| PA14 Triple CIP vs. PA14 EC CIP | -0.657 | -0.635 | -0.022 | >0.9999 |
| PA14 Triple CIP vs. PA14 EC 109 CIP | -0.657 | -0.661 | 0.004 | >0.9999 |
| PA14 Triple CIP vs. PA14 109 CIP | -0.657 | -0.623 | -0.034 | >0.9999 |
| PA14 Triple CIP vs. PA14 109 EC | -0.657 | -0.100 | -0.557 | <0.0001 |
| PA14 Triple CIP vs. PA14 TRIPLE | -0.657 | -0.171 | -0.486 | <0.0001 |
| PA14 EC vs. PA14 109 | -0.038 | -0.045 | 0.007 | >0.9999 |
| PA14 EC vs. PA14 EM 109 | -0.038 | -0.394 | 0.356 | 0.0003 |
| PA14 EC vs. PA14 CIP | -0.038 | -0.459 | 0.421 | <0.0001 |
| PA14 EC vs. PA14 EM 109 CIP | -0.038 | -0.721 | 0.683 | <0.0001 |
| PA14 EC vs. PA14 EM CIP | -0.038 | -0.670 | 0.631 | <0.0001 |
| PA14 EC vs. PA14 EM EC CIP | -0.038 | -0.672 | 0.634 | <0.0001 |
| PA14 EC vs. PA14 EC CIP | -0.038 | -0.635 | 0.597 | <0.0001 |
| PA14 EC vs. PA14 EC 109 CIP | -0.038 | -0.661 | 0.623 | <0.0001 |
| PA14 EC vs. PA14 109 CIP | -0.038 | -0.623 | 0.584 | <0.0001 |
| PA14 EC vs. PA14 109 EC | -0.038 | -0.100 | 0.061 | >0.9999 |
| PA14 EC vs. PA14 TRIPLE | -0.038 | -0.171 | 0.132 | 0.9541 |
| PA14 109 vs. PA14 EM 109 | -0.045 | -0.394 | 0.349 | 0.0004 |
| PA14 109 vs. PA14 CIP | -0.045 | -0.459 | 0.414 | <0.0001 |
| PA14 109 vs. PA14 EM 109 CIP | -0.045 | -0.721 | 0.676 | <0.0001 |
| PA14 109 vs. PA14 EM CIP | -0.045 | -0.670 | 0.624 | <0.0001 |
| PA14 109 vs. PA14 EM EC CIP | -0.045 | -0.672 | 0.627 | <0.0001 |
| PA14 109 vs. PA14 EC CIP | -0.045 | -0.635 | 0.590 | <0.0001 |
| PA14 109 vs. PA14 EC 109 CIP | -0.045 | -0.661 | 0.616 | <0.0001 |
| PA14 109 vs. PA14 109 CIP | -0.045 | -0.623 | 0.577 | <0.0001 |
| PA14 109 vs. PA14 109 EC | -0.045 | -0.100 | 0.054 | >0.9999 |
| PA14 109 vs. PA14 TRIPLE | -0.045 | -0.171 | 0.125 | 0.9749 |
| PA14 EM 109 vs. PA14 CIP | -0.394 | -0.459 | 0.065 | >0.9999 |
| PA14 EM 109 vs. PA14 EM 109 CIP | -0.394 | -0.721 | 0.327 | 0.0013 |
| PA14 EM 109 vs. PA14 EM CIP | -0.394 | -0.670 | 0.275 | 0.0177 |
| PA14 EM 109 vs. PA14 EM EC CIP | -0.394 | -0.672 | 0.278 | 0.0156 |
| PA14 EM 109 vs. PA14 EC CIP | -0.394 | -0.635 | 0.241 | 0.0814 |
| PA14 EM 109 vs. PA14 EC 109 CIP | -0.394 | -0.661 | 0.267 | 0.0265 |
| PA14 EM 109 vs. PA14 109 CIP | -0.394 | -0.623 | 0.228 | 0.1313 |
| PA14 EM 109 vs. PA14 109 EC | -0.394 | -0.100 | -0.295 | 0.0069 |
| PA14 EM 109 vs. PA14 TRIPLE | -0.394 | -0.171 | -0.224 | 0.1557 |
| PA14 CIP vs. PA14 EM 109 CIP | -0.459 | -0.721 | 0.262 | 0.0327 |
| PA14 CIP vs. PA14 EM CIP | -0.459 | -0.670 | 0.211 | 0.2421 |
| PA14 CIP vs. PA14 EM EC CIP | -0.459 | -0.672 | 0.213 | 0.2221 |
| PA14 CIP vs. PA14 EC CIP | -0.459 | -0.635 | 0.176 | 0.592 |
| PA14 CIP vs. PA14 EC 109 CIP | -0.459 | -0.661 | 0.202 | 0.3152 |
| PA14 CIP vs. PA14 109 CIP | -0.459 | -0.623 | 0.164 | 0.7279 |
| PA14 CIP vs. PA14 109 EC | -0.459 | -0.100 | -0.359 | 0.0002 |
| PA14 CIP vs. PA14 TRIPLE | -0.459 | -0.171 | -0.288 | 0.0094 |
| PA14 EM 109 CIP vs. PA14 EM CIP | -0.721 | -0.670 | -0.051 | >0.9999 |
| PA14 EM 109 CIP vs. PA14 EM EC CIP | -0.721 | -0.672 | -0.049 | >0.9999 |
| PA14 EM 109 CIP vs. PA14 EC CIP | -0.721 | -0.635 | -0.086 | >0.9999 |
| PA14 EM 109 CIP vs. PA14 EC 109 CIP | -0.721 | -0.661 | -0.060 | >0.9999 |
| PA14 EM 109 CIP vs. PA14 109 CIP | -0.721 | -0.623 | -0.098 | 0.9992 |
| PA14 EM 109 CIP vs. PA14 109 EC | -0.721 | -0.100 | -0.621 | <0.0001 |
| PA14 EM 109 CIP vs. PA14 TRIPLE | -0.721 | -0.171 | -0.550 | <0.0001 |
| PA14 EM CIP vs. PA14 EM EC CIP | -0.670 | -0.672 | 0.003 | >0.9999 |
| PA14 EM CIP vs. PA14 EC CIP | -0.670 | -0.635 | -0.035 | >0.9999 |
| PA14 EM CIP vs. PA14 EC 109 CIP | -0.670 | -0.661 | -0.009 | >0.9999 |
| PA14 EM CIP vs. PA14 109 CIP | -0.670 | -0.623 | -0.047 | >0.9999 |
| PA14 EM CIP vs. PA14 109 EC | -0.670 | -0.100 | -0.570 | <0.0001 |
| PA14 EM CIP vs. PA14 TRIPLE | -0.670 | -0.171 | -0.499 | <0.0001 |
| PA14 EM EC CIP vs. PA14 EC CIP | -0.672 | -0.635 | -0.037 | >0.9999 |
| PA14 EM EC CIP vs. PA14 EC 109 CIP | -0.672 | -0.661 | -0.011 | >0.9999 |
| PA14 EM EC CIP vs. PA14 109 CIP | -0.672 | -0.623 | -0.050 | >0.9999 |
| PA14 EM EC CIP vs. PA14 109 EC | -0.672 | -0.100 | -0.573 | <0.0001 |
| PA14 EM EC CIP vs. PA14 TRIPLE | -0.672 | -0.171 | -0.502 | <0.0001 |
| PA14 EC CIP vs. PA14 EC 109 CIP | -0.635 | -0.661 | 0.026 | >0.9999 |
| PA14 EC CIP vs. PA14 109 CIP | -0.635 | -0.623 | -0.012 | >0.9999 |
| PA14 EC CIP vs. PA14 109 EC | -0.635 | -0.100 | -0.535 | <0.0001 |
| PA14 EC CIP vs. PA14 TRIPLE | -0.635 | -0.171 | -0.464 | <0.0001 |
| PA14 EC 109 CIP vs. PA14 109 CIP | -0.661 | -0.623 | -0.038 | >0.9999 |
| PA14 EC 109 CIP vs. PA14 109 EC | -0.661 | -0.100 | -0.561 | <0.0001 |
| PA14 EC 109 CIP vs. PA14 TRIPLE | -0.661 | -0.171 | -0.490 | <0.0001 |
| PA14 109 CIP vs. PA14 109 EC | -0.623 | -0.100 | -0.523 | <0.0001 |
| PA14 109 CIP vs. PA14 TRIPLE | -0.623 | -0.171 | -0.452 | <0.0001 |
| PA14 109 EC vs. PA14 TRIPLE | -0.100 | -0.171 | 0.071 | >0.9999 |

**Table S2:** Statistical evaluation of biofilm time-kill analyses

| **PAO1** | | | | | |
| --- | --- | --- | --- | --- | --- |
| **Comparison** | **Mean 1** | **Mean 2** | **Mean Diff.** | **SE of diff.** | **Adjusted P Value** |
| Growth Control vs. EM | -0.889 | -2.587 | 1.698 | 0.399 | 0.0068 |
| Growth Control vs. EC | -0.889 | -1.996 | 1.107 | 0.399 | 0.1936 |
| Growth Control vs. 109 | -0.889 | -1.005 | 0.116 | 0.399 | >0.9999 |
| Growth Control vs. CIP | -0.889 | -2.32 | 1.431 | 0.399 | 0.0349 |
| Growth Control vs. EM + CIP | -0.889 | -1.866 | 0.977 | 0.399 | 0.3369 |
| Growth Control vs. EC + CIP | -0.889 | -1.146 | 0.257 | 0.399 | 0.9996 |
| Growth Control vs. 109 + CIP | -0.889 | -2.417 | 1.528 | 0.399 | 0.0196 |
| Growth Control vs. EM + EC + 109 | -0.889 | -1.387 | 0.498 | 0.399 | 0.9571 |
| Growth Control vs. EM + EC + 109 + CIP | -0.889 | -1.154 | 0.265 | 0.399 | 0.9995 |
| EM vs. EC | -2.587 | -1.996 | -0.591 | 0.3258 | 0.7214 |
| EM vs. 109 | -2.587 | -1.005 | -1.582 | 0.3258 | 0.0014 |
| EM vs. CIP | -2.587 | -2.32 | -0.267 | 0.3258 | 0.9976 |
| EM vs. EM + CIP | -2.587 | -1.866 | -0.721 | 0.3258 | 0.4714 |
| EM vs. EC + CIP | -2.587 | -1.146 | -1.441 | 0.3258 | 0.0044 |
| EM vs. 109 + CIP | -2.587 | -2.417 | -0.17 | 0.3258 | >0.9999 |
| EM vs. EM + EC + 109 | -2.587 | -1.387 | -1.2 | 0.3258 | 0.0278 |
| EM vs. EM + EC + 109 + CIP | -2.587 | -1.154 | -1.433 | 0.3258 | 0.0047 |
| EC vs. 109 | -1.996 | -1.005 | -0.991 | 0.3258 | 0.1152 |
| EC vs. CIP | -1.996 | -2.32 | 0.324 | 0.3258 | 0.9902 |
| EC vs. EM + CIP | -1.996 | -1.866 | -0.13 | 0.3258 | >0.9999 |
| EC vs. EC + CIP | -1.996 | -1.146 | -0.85 | 0.3258 | 0.2595 |
| EC vs. 109 + CIP | -1.996 | -2.417 | 0.421 | 0.3258 | 0.9473 |
| EC vs. EM + EC + 109 | -1.996 | -1.387 | -0.609 | 0.3258 | 0.688 |
| EC vs. EM + EC + 109 + CIP | -1.996 | -1.154 | -0.842 | 0.3258 | 0.2705 |
| 109 vs. CIP | -1.005 | -2.32 | 1.315 | 0.3258 | 0.0118 |
| 109 vs. EM + CIP | -1.005 | -1.866 | 0.861 | 0.3258 | 0.2449 |
| 109 vs. EC + CIP | -1.005 | -1.146 | 0.141 | 0.3258 | >0.9999 |
| 109 vs. 109 + CIP | -1.005 | -2.417 | 1.412 | 0.3258 | 0.0056 |
| 109 vs. EM + EC + 109 | -1.005 | -1.387 | 0.382 | 0.3258 | 0.9708 |
| 109 vs. EM + EC + 109 + CIP | -1.005 | -1.154 | 0.149 | 0.3258 | >0.9999 |
| CIP vs. EM + CIP | -2.32 | -1.866 | -0.454 | 0.3258 | 0.9192 |
| CIP vs. EC + CIP | -2.32 | -1.146 | -1.174 | 0.3258 | 0.0335 |
| CIP vs. 109 + CIP | -2.32 | -2.417 | 0.097 | 0.3258 | >0.9999 |
| CIP vs. EM + EC + 109 | -2.32 | -1.387 | -0.933 | 0.3258 | 0.1637 |
| CIP vs. EM + EC + 109 + CIP | -2.32 | -1.154 | -1.166 | 0.3258 | 0.0355 |
| EM + CIP vs. EC + CIP | -1.866 | -1.146 | -0.72 | 0.3258 | 0.4733 |
| EM + CIP vs. 109 + CIP | -1.866 | -2.417 | 0.551 | 0.3258 | 0.791 |
| EM + CIP vs. EM + EC + 109 | -1.866 | -1.387 | -0.479 | 0.3258 | 0.8926 |
| EM + CIP vs. EM + EC + 109 + CIP | -1.866 | -1.154 | -0.712 | 0.3258 | 0.4884 |
| EC + CIP vs. 109 + CIP | -1.146 | -2.417 | 1.271 | 0.3258 | 0.0164 |
| EC + CIP vs. EM + EC + 109 | -1.146 | -1.387 | 0.241 | 0.3258 | 0.9989 |
| EC + CIP vs. EM + EC + 109 + CIP | -1.146 | -1.154 | 0.008 | 0.3258 | >0.9999 |
| 109 + CIP vs. EM + EC + 109 | -2.417 | -1.387 | -1.03 | 0.3258 | 0.0898 |
| 109 + CIP vs. EM + EC + 109 + CIP | -2.417 | -1.154 | -1.263 | 0.3258 | 0.0175 |
| EM + EC + 109 vs. EM + EC + 109 + CIP | -1.387 | -1.154 | -0.233 | 0.3258 | 0.9992 |
| **PA14** | | | | | |
| **Comparison** | **Mean 1** | **Mean 2** | **Mean Diff.** | **SE of diff.** | **Adjusted P Value** |
| Growth Control vs. EM | -0.403 | -0.698 | 0.295 | 0.2815 | 0.9862 |
| Growth Control vs. EC | -0.403 | -0.523 | 0.12 | 0.2815 | >0.9999 |
| Growth Control vs. 109 | -0.403 | -1.434 | 1.031 | 0.2815 | 0.0276 |
| Growth Control vs. CIP | -0.403 | -1.728 | 1.325 | 0.2815 | 0.0019 |
| Growth Control vs. EM + CIP | -0.403 | -0.78 | 0.377 | 0.2815 | 0.9358 |
| Growth Control vs. EC + CIP | -0.403 | -1.638 | 1.235 | 0.2815 | 0.0044 |
| Growth Control vs. 109 + CIP | -0.403 | -2.141 | 1.738 | 0.2815 | <0.0001 |
| Growth Control vs. EM + EC + 109 | -0.403 | -0.471 | 0.068 | 0.2815 | >0.9999 |
| Growth Control vs. EM + EC + 109 + CIP | -0.403 | -3.176 | 2.773 | 0.2815 | <0.0001 |
| EM vs. EC | -0.698 | -0.523 | -0.175 | 0.2815 | 0.9997 |
| EM vs. 109 | -0.698 | -1.434 | 0.736 | 0.2815 | 0.2543 |
| EM vs. CIP | -0.698 | -1.728 | 1.03 | 0.2815 | 0.0278 |
| EM vs. EM + CIP | -0.698 | -0.78 | 0.082 | 0.2815 | >0.9999 |
| EM vs. EC + CIP | -0.698 | -1.638 | 0.94 | 0.2815 | 0.0589 |
| EM vs. 109 + CIP | -0.698 | -2.141 | 1.443 | 0.2815 | 0.0006 |
| EM vs. EM + EC + 109 | -0.698 | -0.471 | -0.227 | 0.2815 | 0.9979 |
| EM vs. EM + EC + 109 + CIP | -0.698 | -3.176 | 2.478 | 0.2815 | <0.0001 |
| EC vs. 109 | -0.523 | -1.434 | 0.911 | 0.2815 | 0.0741 |
| EC vs. CIP | -0.523 | -1.728 | 1.205 | 0.2815 | 0.0058 |
| EC vs. EM + CIP | -0.523 | -0.78 | 0.257 | 0.2815 | 0.9948 |
| EC vs. EC + CIP | -0.523 | -1.638 | 1.115 | 0.2815 | 0.0132 |
| EC vs. 109 + CIP | -0.523 | -2.141 | 1.618 | 0.2815 | 0.0001 |
| EC vs. EM + EC + 109 | -0.523 | -0.471 | -0.052 | 0.2815 | >0.9999 |
| EC vs. EM + EC + 109 + CIP | -0.523 | -3.176 | 2.653 | 0.2815 | <0.0001 |
| 109 vs. CIP | -1.434 | -1.728 | 0.294 | 0.2815 | 0.9865 |
| 109 vs. EM + CIP | -1.434 | -0.78 | -0.654 | 0.2815 | 0.4038 |
| 109 vs. EC + CIP | -1.434 | -1.638 | 0.204 | 0.2815 | 0.9991 |
| 109 vs. 109 + CIP | -1.434 | -2.141 | 0.707 | 0.2815 | 0.3024 |
| 109 vs. EM + EC + 109 | -1.434 | -0.471 | -0.963 | 0.2815 | 0.0489 |
| 109 vs. EM + EC + 109 + CIP | -1.434 | -3.176 | 1.742 | 0.2815 | <0.0001 |
| CIP vs. EM + CIP | -1.728 | -0.78 | -0.948 | 0.2815 | 0.0552 |
| CIP vs. EC + CIP | -1.728 | -1.638 | -0.09 | 0.2815 | >0.9999 |
| CIP vs. 109 + CIP | -1.728 | -2.141 | 0.413 | 0.2815 | 0.8944 |
| CIP vs. EM + EC + 109 | -1.728 | -0.471 | -1.257 | 0.2815 | 0.0036 |
| CIP vs. EM + EC + 109 + CIP | -1.728 | -3.176 | 1.448 | 0.2815 | 0.0006 |
| EM + CIP vs. EC + CIP | -0.78 | -1.638 | 0.858 | 0.2815 | 0.1109 |
| EM + CIP vs. 109 + CIP | -0.78 | -2.141 | 1.361 | 0.2815 | 0.0013 |
| EM + CIP vs. EM + EC + 109 | -0.78 | -0.471 | -0.309 | 0.2815 | 0.9812 |
| EM + CIP vs. EM + EC + 109 + CIP | -0.78 | -3.176 | 2.396 | 0.2815 | <0.0001 |
| EC + CIP vs. 109 + CIP | -1.638 | -2.141 | 0.503 | 0.2815 | 0.7377 |
| EC + CIP vs. EM + EC + 109 | -1.638 | -0.471 | -1.167 | 0.2815 | 0.0082 |
| EC + CIP vs. EM + EC + 109 + CIP | -1.638 | -3.176 | 1.538 | 0.2815 | 0.0002 |
| 109 + CIP vs. EM + EC + 109 | -2.141 | -0.471 | -1.67 | 0.2815 | <0.0001 |
| 109 + CIP vs. EM + EC + 109 + CIP | -2.141 | -3.176 | 1.035 | 0.2815 | 0.0267 |
| EM + EC + 109 vs. EM + EC + 109 + CIP | -0.471 | -3.176 | 2.705 | 0.2815 | <0.0001 |
